# New Glutamine-Containing Azaphilone Alkaloids from Deep-Sea-Derived Fungus *Chaetomium globosum* HDN151398

**DOI:** 10.3390/md17050253

**Published:** 2019-04-28

**Authors:** Chunxiao Sun, Xueping Ge, Shah Mudassir, Luning Zhou, Guihong Yu, Qian Che, Guojian Zhang, Jixing Peng, Qianqun Gu, Tianjiao Zhu, Dehai Li

**Affiliations:** 1 Key Laboratory of Marine Drugs, Chinese Ministry of Education, School of Medicine and Pharmacy, Ocean University of China, Qingdao 266003, China; sunchunxiao93@163.com (C.S.); 15610568273@163.com (X.G.); s84mudassir@gmail.com (S.M.); 18895692529@163.com (L.Z.); cheqian064@ouc.edu.cn (Q.C.); zhangguojian@ouc.edu.cn (G.Z.); guqianq@ouc.edu.cn (Q.G.); 2 Laboratory for Marine Drugs and Bioproducts of Qingdao National Laboratory for Marine Science and Technology, Qingdao 266237, China; Yuguihong1990@126.com; 3 Key Laboratory of Testing and Evaluation for Aquatic Product Safety and Quality, Ministry of Agriculture and Rural Affairs, P. R. China; Yellow Sea Fisheries Research Institute, Chinese Academy of Fishery Sciences, Qingdao 266071, China; pengjixing1987@163.com; 4 Open Studio for Druggability Research of Marine Natural Products, Pilot National Laboratory for Marine Science and Technology, Qingdao 266237, China

**Keywords:** *Chaetomium globosum*, azaphilone alkaloids, cytotoxicity, deep-sea-derived fungus

## Abstract

Three new azaphilone alkaloids containing glutamine residues, namely *N*-glutarylchaetoviridins A–C (**1**–**3**), together with two related compounds (**4** and **5**) were isolated from the extract of *Chaetomium globosum* HDN151398, a fungus isolated from a deep-sea sediment sample collected in South China Sea. Their structures were elucidated on the basis of extensive 1D and 2D NMR as well as HRESIMS spectroscopic data and chemical analysis. *N*-glutarylchaetoviridins A–C (**1**–**3**) represent the first class of chaetoviridins characterized by embedded glutamate residues. Amino acids incubation experiments produced five azaphilone laden different amino acids residues (**6**–**10**) which indicated that this method can enhanced the structural diversity of this strain by culturing with amino acids. Cytotoxicity of the isolated compounds were evaluated against a panel of human cancer cell lines.

## 1. Introduction

Deep-sea-derived microorganisms have proven to be a prolific source of secondary metabolites with an ample variety of captivating chemical structures and diverse pharmacological properties [1,2]. In our recent search for bioactive secondary metabolites from marine-derived fungi, a fungal strain *Chaetomium globosum* HDN151398, isolated from a deep-sea sediment sample (depth 2476 m) collected from South China Sea, was selected for its intriguing HPLC-UV profile (Figure S1) and significant crude extract cytotoxic activity (69% inhibition of K562 cells at the concentration of 100 μg/mL). Chemical investigation of the organic extract of the fungus led to the isolation of three new glutamine-containing azaphilone alkaloids, *N*-glutarylchaetoviridins A–C (**1**–**3**) together with two known chaetoviridins (**4** and **5**).

**Figure 1:**
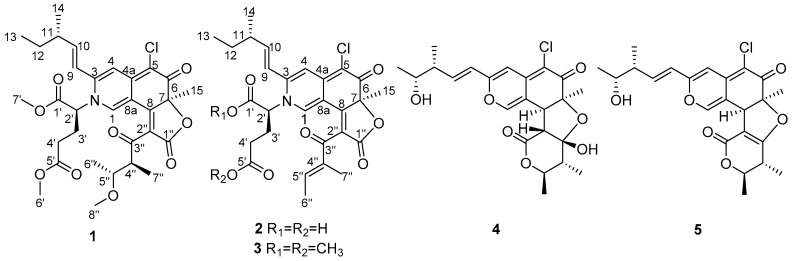
Structures of **1**–**5**.

Azaphilones are a family of structurally erratic fungal pigmented polyketides with pyrone-quinone structures containing a highly oxygenated bicyclic core and a chiral quaternary center [3,4]. The oxygen atom in the pyran chromophore of the azaphilones could be biosynthetically replaced by nitrogen atom in the presence of primary amines and the colour of the pigment shifted to red accordingly [4]. Recently, azaphilones have been recognized as a unique family of secondary metabolites with diverse bioactivities including antimicrobial [5], cytotoxic [6], anti-inflammatory [7] and other activities [8,9,10], which have provoked enormous attention of scientists for biosynthesis [11] and chemical synthesis studies [12]. In the present work, we report the isolation, structure elucidation and biological activities of the previously unreported azaphilones (**1**–**3**) (Figure 1) from the strain *Chaetomium globosum* HDN151398 as well as the incubation experiments with different amino acids to produce more diverse analogues.

## 2. Results and Discussion

**Figure 2:**
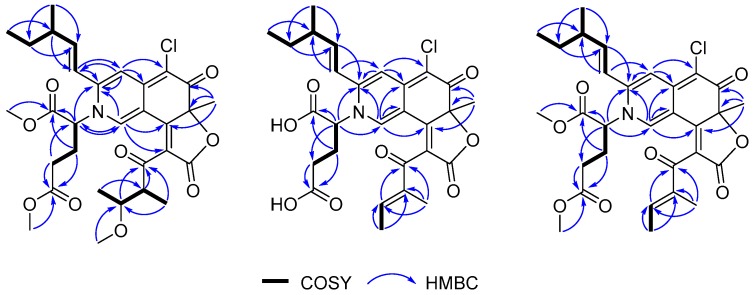
Key HMBC and ^1^H-^1^H COSY correlations for **1**−**3**.

Compound **1** was isolated as a dark red powder with the molecular formula C_31_H_38_O_9_NCl determined by the (+)-HRESIMS peak at *m/z* 604.2308 [M + H]^+^ (calcd. for C_31_H_39_O_9_NCl, 604.2308), indicating 13 degrees of unsaturation. An isotopic peak ratio of 3:1 for [M + H]^+^:[M + H + 2]^+^ was observed, indicating the presence of a single chlorine atom in the molecule. The infrared (IR) absorption at 1717 cm^−1^ indicated the presence of the carbonyl functionality. The ^1^H NMR data (Table 1) of **1** showed eight methyls [*δ*_H_ 0.95 (3H, t, *J* = 7.4 Hz, H-13), 1.13 (3H, d, *J* = 6.7 Hz, H-14), 1.73 (3H, s, H-15), 3.72 (3H, s, H-6′), 3.83 (3H, s, H-7′), 1.04 (3H, d, *J* = 6.3 Hz, H-6″), 1.07 (3H, d, *J* = 6.8 Hz, H-7″), 2.93 (3H, s, H-8″)], and four olefinic protons [*δ*_H_ 8.48 (1H, s, H-1), 6.84 (1H, s, H-4), 6.25 (1H, d, *J* = 15.4 Hz, H-9), 6.40 (1H, dd, *J* = 7.5, 15.4 Hz, H-10)]. The ^13^C NMR data (Table 1), assigned by the aid of DEPT and HSQC spectra, displayed the resonances of eight methyl (*δ*_C_ 8.4, 11.7, 16.1, 16.1, 26.6, 52.1, 53.6, 56.2), three methylene (*δ*_C_ 27.0, 29.0, 29.2), eight methine (*δ*_C_ 39.0, 49.3, 61.9, 77.7, 111.1, 119.0, 136.9, 150.3), and twelve nonprotonated carbons (*δ*_C_ 88.5, 101.2, 112.4, 125.0, 144.5, 148.4, 165.4, 167.9, 168.4, 172.2, 182.1, 199.3). Careful comparison of the ^13^C NMR data of **1** and those of chaetoviridin A [13] revealed that they share a similar pyrone-quinone-containing skeleton. The main differences between **1** and chaetoviridin A were the chemical shifts at C-1 (*δ*_C_ 136.9 versus *δ*_C_ 151.5) and C-3 (*δ*_C_ 148.4 versus *δ*_C_ 157.1), and a group of extra resonances in **1** which were attributed to a methylated glutamic acid moiety. The pyrone-quinone core structure was further verified by the ^1^H-^1^H COSY cross peaks from H-6″ to H-5″, from H-5″ to H-4″, from H-4″ to H-7″, from H-13 to H-12, from H-12 to H-11, from H-11 to H-10, from H-10 to H-9 and from H-14 to H-11 as well as HMBC correlations from H-1 to C-8 and C-3, from H-9 to C-3 and C-4, from H-4 to C-5 and C-4a, from H-15 to C-6, C-7, and C-8 and from H-4″ to C-3″and C-2″(Figure 2). The methylated glutamic acid moiety was deduced by the COSY correlations from H-2′ to H-3′and from H-3′ to H-4′ as well as the HMBC correlations from H-6′ and H-4′ to C-5′, from H-7′ and H-2′ to C-1′. Based on the variation between the chemical shifts at C-1 and C-3 and taking the molecular formula into account, a nitrogen atom, instead of an oxygen atom, was placed in position 2. Further HMBC cross peaks from H-2′ to C-1 (*δ* 136.9) and C-3 (*δ* 148.4) (Figure 2) attached the dimethylglutarate moiety to N-2 of 1,4-hydropyridine-quinone scaffold moiety. As this compound has never been previously reported, it was named *N*-glutarylchaetoviridin A.

**Figure 3:**
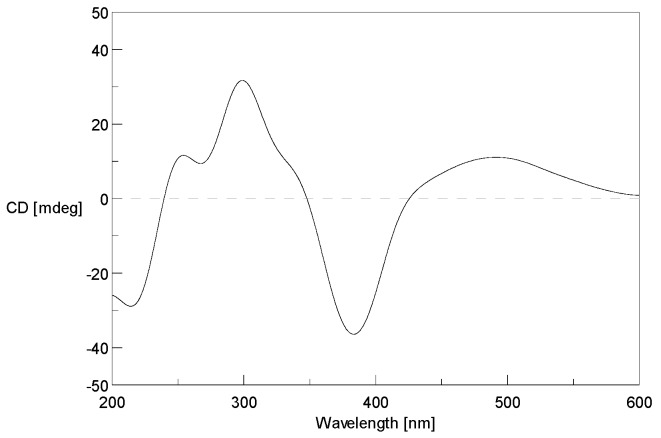
Experimental electronic circular dichroism (ECD) spectrum of **1** in methanol.

**Figure 4:**
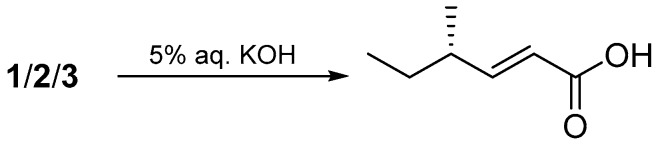
Alkaline degradation of **1**–**3**.

The geometrical configuration of the double bond between C-9 and C-10 was inferred to be *trans* from the coupling constants of the olefinic protons (*J*_9,10_ = 15.5 Hz). The relative configuration of **1** was determined based on a combination of NOESY correlations and comparison of its NMR data with those of chaetoviridin A. The stereochemistry of **1** established based on the NOESY correlations from H-1 to H-4″, from H-1 to H-6″, and from H-8″ to H-7″, the similar electronic circular dichroism (ECD) curves of **1** (Figure 3) and chaetoviridin A [13,14], and the co-isolation of biogenetically related compounds **4** and **5** which also shared the same chiral centres. The absolute configuration of C-7 and C-11 were further confirmed by Steyn and Vleggaar’s method [15] and the degradation of **1** [16], respectively. The ECD spectrum of **1** (Δ*ε*387 −10.1; Figure 3) revealed that the absolute configuration of C-7 is *S* according to Steyn and Vleggaar’s CD method [15]. Compound **1** was degraded by 5% potassium hydroxide to afford a carboxylic acid (Figure 4) which was identified as (4*S*)-2*E*-4-methylhex-2-enoic acid by comparison of spectral data and specific optical rotation with the authentic sample. The configuration of the _L_-glutamate moiety in **1** was determined by the advanced Marfey’s method [17] by comparison of the retention time and mass data of the hydrolysis product with those of d/l-glutamate standards by HPLC (Figure S30). Accordingly, the absolute configuration of **1** was concluded to be 7*S*, 11*S*, 2′*S*, 4″*S*, and 5″*R*, respectively.

Compound **2** was isolated as a dark red powder with the molecular formula C_28_H_30_O_8_NCl determined by the (+)-HRESIMS *m/z* 544.1731 [M + H]^+^ (calcd. for C_28_H_31_O_8_NCl, 544.1733), requiring 14 degrees of unsaturation. The isotopic peak [M + H]^+^:[M + H + 2]^+^=3:1 was observed, indicating the presence of a single chlorine atom in the molecule. The IR spectrum displayed absorption bands for carbonyl (1684 and 1761 cm^−1^) functionalities. The ^1^H NMR data (Table 1) of **2** showed five methyls [*δ*_H_ 0.96 (3H, t, *J* = 7.5 Hz, H-13), 1.13 (3H, d, *J* = 6.7 Hz, H-14), 1.69 (3H, s, H-15), 1.89 (3H, d, *J* = 7.0 Hz, H-6″), 1.87 (3H, s, H-7″)], and five olefinic protons [*δ*_H_ 7.76 (1H, s, H-1), 6.90 (1H, s, H-4), 6.46 (1H, d, *J* = 14.2 Hz, H-9), 6.41 (1H, m, H-10), 6.64 (1H, q, *J* = 6.9 Hz, H-5″)]. The ^13^C NMR (Table 2), in combination with DEPT and HSQC spectra, displayed the resonances of five methyl (*δ*_C_ 9.2, 10.6, 14.0, 17.9, 25.0), three methylene (*δ*_C_ 26.8, 28.6, 29.2), seven methine (*δ*_C_ 38.9, 62.7, 110.6, 119.7, 136.3, 146.7, 150.1), thirteen quaternary (*δ*_C_ 88.3, 99.4, 112.4, 124.4, 138.0, 145.9, 150.4, 161.6, 168.3, 169.4, 173.8, 181.8, 190.6) carbons. The ^1^H and ^13^C NMR data of **2** were very similar to those of **1**, while the main differences were the absence of three methyl and one proton signals (*δ*_H_ 3.83, 3.72, 2.93, and 3.71, respectively) and the downfield shift of H-7″ (*δ* 1.87), H-6″ (*δ* 1.89) and H-5″ (*δ* 6.64), suggesting that the single bond between C-4″ (*δ* 138.0) and C-5″ (*δ* 146.7) was oxidized to a double bond. This postulation was confirmed by COSY correlations from H-5″ to H-6″ and HMBC cross peaks from H-7″to C-3″and C-4″, and from H-5″ to C-3″. Further 2D NMR analysis (Figure 2) verified the planar structure as shown in Figure 1 and we named it *N*-glutarylchaetoviridin B.

**Figure 5:**
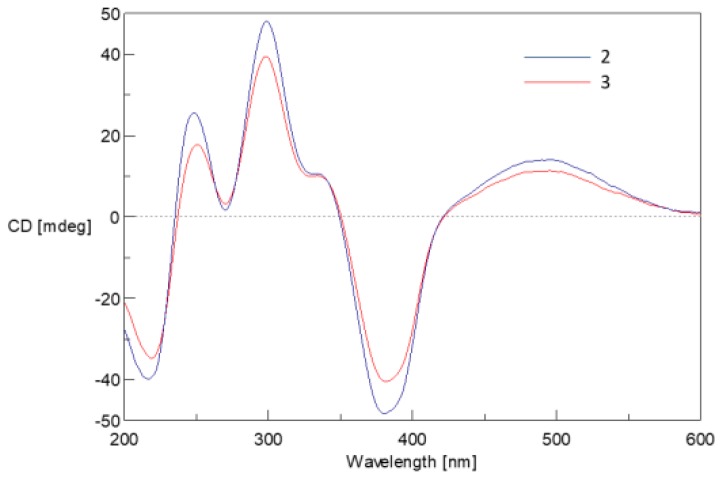
Experimental ECD spectra of **2** and **3** in methanol.

The coupling constants of the olefinic protons (*J*_9,10_ = 14.2 Hz) indicated the *trans* configuration of the double bond (*Δ*_9,10_). The NOESY correlations between H-6″/H-7″ demonstrated that the double bond between C-4″ and C-5″ was in *E* configuration. The amino acid residue in **2** was identified as l-glutamate by the advanced Marfey’s method [17]. The CD spectrum of **2** (*Δ*ε387 −10.8; Figure 5) revealed the 7*S* absolute configuration according to Steyn and Vleggaar’s CD method [15]. The absolute configuration at C-11 was determined as *S* by the degradation of **2**. Thus, the absolute configurations of C-7, 11 and 2′ of **2** were assigned as 7*S*, 11*S*, and 2′*S*.

Compound **3** was isolated as a dark red powder with the molecular formula C_30_H_34_O_8_NCl determined by the (+)-HRESIMS *m/z* 572.2050 [M + H]^+^ (calcd. for C_30_H_35_O_8_NCl, 572.2046), indicating 14 degrees of unsaturation. Comparison of the 1D NMR data of **3** with those of **2**, it was found that there are two methoxy groups [*δ*_H_ 3.67 (3H, s, H-6′), 3.77 (3H, s, H-7′); *δ*_C_ 52.1, 53.4] in **3**. Mass spectrometric data as well as the key HMBC correlations from H-6′ (*δ* 3.67) to C-5′ (*δ* 172.1) and H-7′ (*δ* 3.77) to C-1′ (*δ* 168.3) confirmed that **3** is a 6′,7′-dimethoxyl analogue of **2**, and was named *N*-glutarylchaetoviridin C. As the ECD curve of **3** is very similar to that of **2** (Figure 5) it was concluded that **3** has the same stereochemistry as **2**.

Two previously reported compounds, chaetomugilin A (**4**) [18] and chaetomugilin C (**5**) [18] were also isolated and their identity was proved by comparison of their NMR and MS data with those reported in the literature.

Compounds **1**–**5** were evaluated for their cytotoxic activity against twelve human cancer cell lines including human hepatocellular carcinoma cell line (BEL-7402), human colon cancer cell line (HCT-116), human cervix cancer cell line (HeLa), human hepatic cancer cell line (L-02), human gastric cancer cell line (MGC-803), human ovarian cancer cell line (HO8910), human neuroblastoma cell line (SH-SY5Y), human lung adenocarcinoma cell line (NCI-H1975), human glioblastoma cell line (U87), human breast cancer cell line (MDA-MB-231), human kidney cancer cell line (K562) and human promyelocytic leukemia cell line (HL-60) (Table S1). Compounds **3**, **4,** and **5** showed a broad spectrum of cytotoxic activity. Among them, **3** showed significant cytotoxic activity against MGC-803 and HO8910 with IC_50_ values of 6.6 and 9.7 µM, respectively.

**Figure 6:**
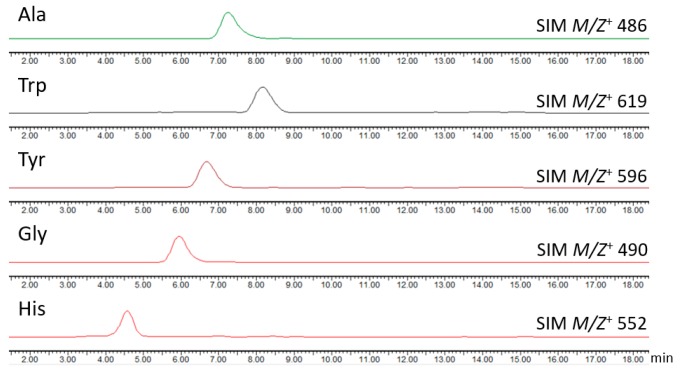
LC/MS analysis of the metabolic extracts from the HDN151398 by incubation with different amino acids.

**Figure 7:**
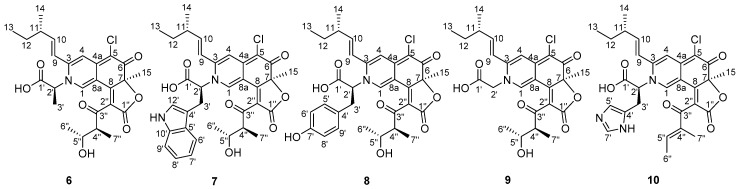
Structures of **6**–**10**.

Inspired by the fact that azaphilones have a capacity to incorporate amino acids, five different amino acids (l-tryptophan, l-tyrosine, l-histidine, l-alanine, l-glycine) were added to the culture medium in order to produce more diverse analogues. All the molecular ion peaks of the proposed structures could be easily detected by LC-MS (Figure 6). Furthermore, the structures of **6**–**10** (Figure 7) were further confirmed by both (+)-HRESIMS and NMR data (Table 3, Figures S35–S44). Consequently, these results further validate the property of azaphilones to combine with amino acids and to produce more diverse compounds.

## 3. Materials and Methods

### 3.1. General Experimental Procedures

Optical rotations were obtained on a JASCO P-1020 (JASCO Corporation, Tokyo, Japan) digital polarimeter. UV spectra were recorded on Waters 2487 (Waters Corporation, Milford, MA, USA), while the ECD spectrum were recorded on JASCO J-815 spectropolarimeter (JASCO Corporation, Tokyo, Japan). ^1^H NMR, ^13^C NMR, DEPT and 2D NMR spectra were recorded on an Agilent 500 MHz DD2 spectrometer (Agilent Technologies Inc., Santa Clara, CA, USA). HRESIMS and ESIMS spectra were obtained using a Thermo Scientific LTQ Orbitrap XL mass spectrometer (Thermo Fisher Scientific, Waltham, MA, USA) on positive ionisation mode. Column chromatography (CC) was performed with silica gel (200-300 mesh, Qingdao Marine Chemical Inc., Qingdao, China) and Sephadex LH-20 (Amersham Biosciences, San Francisco, CA, USA). MPLC was performed on a Bona-Agela CHEETAHTM HP100 (Beijing Agela Technologies Co., Ltd., Beijing, China). RP-HPLC was performed on an ODS column (HPLC (YMC-Pack ODS-A, 10 × 250 mm, 5 µm, 3 mL/min)) (YMC Co., Ltd., Kyoto, Japan). LC-MS was performed using an Acquity UPLC H-Class coupled to a SQ Detector 2 mass spectrometer using a BEH C_18_ column (1.7 μm, 2.1 × 50 mm, 1mL/min) (Waters Corporation, Milford, MA, USA).

### 3.2. Fungal Material

The fungal strain was isolated from the sediment sample collected from South China Sea (depth 2476 m, E 117.3957°, N 19.9778°, collected in May, 2017) and identified as *Chaetomium globosum* based on sequencing of the ITS region (GenBank no. MH828376) with 100% similarity. The strain was deposited at the Key Laboratory of Marine Drugs, the Ministry of Education of China, School of Medicine and Pharmacy, Ocean University of China, Qingdao, China.

### 3.3. Fermentation

The fungus was cultured under static condition at room temperature for 30 days in 1 L Erlenmeyer flasks each containing 300 mL of liquid culture medium, composed of glucose (1%), maltose (2%), mannitol (2%), monosodium glutamate (1%), KH_2_PO_4_ (0.05%), MgSO_4_·7H_2_O (0.03%), corn steep liquor (0.1%) and yeast extract (0.3%) after adjusting its pH to 6.5 in natural sea water (collected from JiaoZhou Bay, Qingdao, China).

### 3.4. Isolation

The whole fermentation broth (40 L) was filtered through muslin cloth to separate the supernatant from the mycelia. The supernatant was extracted with EtOAc (3 × 40 L), and the mycelia were homogenized and extracted with MeOH (3 × 10 L). The EtOAc and MeOH solutions of the supernatant and mycelia were combined and evaporated under reduced pressure to give a crude. The extract (30.0 g) was fractioned by VLC of silica gel using a step gradient elution DCM-MeOH (100:0 to 0:100) to give ten fractions (Fr.1 to Fr.10). Fr.6 was further fractioned by MPLC (C-18 ODS) using a step gradient elution of MeOH-H_2_O (5:95 to 100:0) to yield 12 subfractions (Fr.6-1 to Fr.6-12). Fr.6-3, Fr.6-4 and Fr.6-5 were further fractioned by a Sephadex LH-20 column with MeOH to provide five subfractions (Fr.6-3-1 to Fr.6-3-5), six fractions (Fr.6-4-1 to Fr.6-4-6) and four fractions (Fr.6-5-1 to Fr.6-5-4) respectively. Fr.6-4-4, Fr.6-3-3 and Fr.6-5-3 were separated by semi-preparative HPLC eluted with MeOH-H_2_O (65:35) to obtain **1** (9.7 mg, *t*_R_ = 32 min) and **4** (10.0 mg, *t*_R_ = 34 min), MeOH-H_2_O (45:55) to obtain **2** (10.0 mg, *t*_R_ = 40 min), and MeOH-H_2_O (70:30) to obtain **5** (15.0 mg, *t*_R_ = 26 min) and **3** (35.0 mg, *t*_R_ = 27 min), respectively.

### 3.5. Absolute Configuration of Amino Acids

Compounds **1**–**3** were hydrolyzed in 6 N HCl at 60 °C overnight. The solution was dried under a stream of N_2_ and dissolved in H_2_O (100 μL). The acid hydrolysates of **1**−**3** were dissolved in H_2_O (50 μL) separately, and then 0.25 μM FDAA in 100 μL of acetone was added, followed by 1 N NaHCO_3_ (25 μL). The mixtures were heated for 1 h at 43 °C. After cooling to room temperature, the reaction was quenched by the addition of 2 N HCl (25 μL). Finally, the resulting solution was filtered through a small 4.5 μm filter and stored in the freezer until ready for HPLC analysis. Amino acid standards were derivatized with FDAA in a similar manner. The resulting FDAA derivatives of compounds **1**−**3**, l- and d-glutamate were separately analyzed by reversed-phase HPLC (5 × 250 mm YMC C18 column, 5 μm, with a linear gradient of MeCN (A) and 0.05% aqueous TFA (B) from 5% to 55% A over 55 min at a flow rate of 1 mL/min, UV detection at 320 nm). Each chromatographic peak was identified by comparing its retention time with the FDAA derivatives of the l- and d- amino acid standards. The standards gave the following retention times (in min): 40.20 for l-FDAA, 38.61 for d-FDAA, 41.32 for l-Me-FDAA, 39.41 for d-Me-FDAA. The analysis gave retention time (in min) of 41.32, 40.20, and 41.32 (Figures S30 and S31), establishing the *S* configuration for all the glutamic acid residues [17,19].

*N-glutarylchaetoviridin A* (**1**): dark red powder; [*α*]${}_{D}^{20}$ +50 (*c* 0.03, MeOH); IR (KBr) *ν*_max_ 3724, 3649, 2924, 2361, 1717, 1652, 1196, 1027, 669 cm^−1^; UV (MeOH) *λ*max (log *ε*): 215 (3.25), 299 (2.10), 389 (2.04) nm; ECD (2.5 mM, MeOH) λmax (*Δ*ε) 240 (+9.26), 300 (+9.33), 390 (−10.10), 490 (+3.56) nm; ^1^H and ^13^C NMR data see Table 1 and Table 2; (+)-HRESIMS *m/z* 604.2308 [M + H]^+^ (calcd. for C_31_H_39_O_9_NCl, 604.2308).

*N-glutarylchaetoviridin B* (**2**): dark red powder; [*α*]${}_{D}^{20}$ +332 (*c* 0.09, MeOH); IR (KBr) *ν*_max_ 3751, 3420, 1761, 1684, 1485, 1190, 1020, 723 cm^−1^; UV (MeOH) *λ*max (log *ε*): 238 (3.20), 298 (2.04), 391 (1.98) nm; ECD (2.5 mM, MeOH) λmax (*Δ*ε) 220 (+9.24), 245 (+4.32), 300 (+10.31), 390 (−10.79), 490 (+3.96) nm; ^1^H and ^13^C NMR data see Table 1 and Table 2; (+)-HRESIMS *m/z* 544.1731 [M + H]^+^ (calcd. for C_28_H_31_O_8_NCl, 544.1733).

*N-glutarylchaetoviridin C* (**3**): dark red powder; [*α*]${}_{D}^{20}$ +456 (*c* 0.07, MeOH); IR (KBr) *ν*_max_ 3676, 2960, 2362, 1759, 1605, 1489, 1193, 1018, 705 cm^−1^; UV (MeOH) *λ*max (log *ε*): 230 (3.15), 295 (2.02), 393 (2.01) nm; ECD (2.5 mM, MeOH) λmax (*Δ*ε) 220 (+9.24), 245 (+4.12), 300 (+10.25), 390 (−10.66), 490 (+3.62) nm; ^1^H and ^13^C NMR data see Table 1 and Table 2; (+)-HRESIMS *m/z* 572.2050 [M + H]^+^ (calcd. for C_30_H_35_O_8_NCl, 572.2046).

### 3.6. Degradation of 1–3 by Potassium Hydroxide

Compounds **1**–**3** (3.0 mg) were separately dissolved in 5% aq. potassium hydroxide (5 mL) and the reaction mixture was stirred for 3 h at 100 °C. Then, the reaction mixture was extracted with CHCl_3_ (5 mL). The water layer was adjusted to pH 3.0 with 9% sulfuric acid and re-extracted with petroleum ether (5 mL). The organic extract was concentrated to dryness in vacuo. The residue was purified by HPLC using MeCN–H_2_O gradient (30:70 to 100:0 in 45 min) as the eluent to afford (4*S*)-2*E*-4-methylhex-2-enoic acid (0.1 mg, *t*_R_ = 15 min). The physicochemical properties of this carboxylic acid were identical to the authentic sample [16].

### 3.7. Cytotoxicity Assay

Cytotoxic activity of **1**–**5** were evaluated against BEL-7402, HCT-116, HeLa, L-02, MGC-803, HO8910, SH-SY5Y, NCI-H1975, U87, MDA-MB-231 cancer cells by SRB method, K562 and HL-60 by MTT method using adriamycin (ADM) as a positive control. The detailed methodologies for biological testing have been described in our previous report [20]. All of the experiments were carried out in triplicate.

### 3.8. Amino Acid Incubation Experiment

The fungus was cultured and subjected to a large-scale fermentation under the same protocol, stated above in the fermentation section. The only difference is that monosodium glutamate was replaced by five different amino acids. Dried extracts were dissolved in 1 mL of MeOH and analyzed by UPLC-MS (MeCN-H_2_O, 1 mL/min; 0–15 min, 5%–95%; 15–18 min, 100%; 18–20 min, 5%) and further separated by semi-preparative HPLC, eluted with MeCN-H_2_O (40:60) to obtain **6** (2.5 mg, *t*_R_ = 24 min), **7** (2.0 mg, *t*_R_ = 30 min) and **8** (1.0 mg, *t*_R_ = 32 min), MeCN-H_2_O (30:70) to obtain **9** (2.5 mg, *t*_R_ = 18 min), and MeCN-H_2_O (25:75) to obtain **10** (1.5 mg, *t*_R_ = 22 min), respectively.

## 4. Conclusions

In summary, a series of azaphilones (**1**–**5**) were isolated from the deep-sea-derived fungus *C. globosum* HDN151398. Distinguished from the previously reported azaphilone derivatives, **1**–**3** belong to a new class of chaetoviridins which is linked to a glutamate residue, indicating that unique geographical features of deep-sea environment may promote the unique biogenetic and metabolic pathways of the microorganisms in which they inhabit. Compounds **3**, **4,** and **5** showed a broad spectrum of cytotoxicity, among which, **3** was active against MGC-803 and HO8910 with the IC_50_ values of 6.6 and 9.7 µM, respectively. Amino acids feeding experiment showed that it is an effective method to increase structural diversity of azaphilones.

## Figures and Tables

**Table 1:** ^1^H NMR data for **1**–**3** at 500 MHz (*δ* in ppm, *J* in Hz).

No.	1 *^a^*	2 *^b^*	3 *^a^*
1	8.48, s	7.76, s	7.81, s
4	6.84, s	6.90, s	6.72
9	6.25, d (15.4)	6.46, d (14.2)	6.17, d (15.4)
10	6.40, dd (7.5, 15.4)	6.41, m	6.32, dd (7.6, 15.4)
11	2.33, m	2.35, m	2.28, m
12	1.49, m	1.51, m	1.44, m
13	0.95, t (7.4)	0.96, t (7.5)	0.91, t (7.5)
14	1.13, d (6.7)	1.13, d (6.7)	1.08, d (6.7)
15	1.73, s	1.69, s	1.69, s
2′	5.13, t (7.9)	5.26, m	5.05, t (7.3)
3′	2.37, m	2.29, m	2.47, m
	2.68, m	2.57, m	2.30, m
4′	2.47, m	2.44, t (6.6)	2.42, m
	2.57, m	2.49, m	2.56, m
6′	3.72, s		3.67, s
7′	3.83, s		3.77, s
4″	3.71, m		
5″	3.56, m	6.64, q (6.9)	6.55, q (6.3)
6″	1.04, d (6.3)	1.89, d (7.0)	1.87, d (6.9)
7″	1.07, d (6.8)	1.87, s	1.83, s
8″	2.93, s		

*^a^* Measured in CDCl_3_; *^b^* Measured in methanol-*d*_4_.

**Table 2:** ^13^C NMR Data for **1**–**3** at 125 MHz (*δ* in ppm).

No.	1 *^a^*	2 *^b^*	3 *^a^*
1	136.9	136.3	135.4
2			
3	148.4	150.4	148.2
4	111.1	110.6	110.7
4a	144.5	145.9	143.8
5	101.2	99.4	101.6
6	182.1	181.8	182.1
7	88.5	88.3	88.5
8	165.4	161.6	162.9
8a	112.4	112.4	112.3
9	119.0	119.7	119.2
10	150.3	150.1	149.9
11	39.0	38.9	38.9
12	29.0	28.6	29.0
13	11.7	10.6	11.6
14	16.1	17.9	19.0
15	26.6	25.0	26.2
1′	167.9	169.4	168.3
2′	61.9	62.7	61.7
3′	27.0	26.8	27.2
			
4′	29.2	29.2	29.3
			
5′	172.2	173.8	172.1
6′	52.1		52.1
7′	53.6		53.4
1″	168.4	168.3	168.1
2″	125.0	124.4	125.1
3″	199.3	190.6	190.8
4″	49.3	138.0	137.6
5″	77.7	146.7	146.5
6″	16.1	14.0	15.4
7″	8.4	9.2	10.7
8″	56.2		

*^a^* Measured in CDCl_3_. *^b^* Measured in methanol-*d*_4_.

**Table 3:** ^1^H NMR data for **6**–**10** (*δ* in ppm, *J* in Hz).

No.	6 *^b^*^,*d*^	7 *^a^*^,*e*^	8 *^a^*^,*e*^	9 *^a^*^,*e*^	10 *^a^*^,*e*^
1	9.08, s	8.61, s	8.53, s	8.53, s	8.99, s
4	6.87, s	6.42, s	6.49, s	6.78, s	6.58, s
9	6.26, d (15.0)	6.14, d (12.8)	6.28, d (12.8)	6.40, d (15.6)	6.34, d (15.7)
10	6.43, m	6.01, dd (5.9, 12.8)	6.14, m	6.45, dd (7.1, 15.6)	6.25, m
11	2.35, m	2.06, m	2.24, m	2.26, m	2.26, m
12	1.59, m	1.26, m	1.39, m	1.39, m	1.40, m
13	0.96, t (6.6)	0.76, t (6.2)	0.86, t (6.2)	0.83, t (7.4)	0.85, t (7.4)
14	1.14, ov. *^c^*	0.98, d (5.6)	0.89, d (5.0)	1.02, d (6.7)	1.03, d (6.7)
15	1.93, s	1.43, s	1.54, s	1.56, s	1.60, s
2′	5.08, m	5.53, dd (4.1, 8.5)	5.49, dd (4.3, 8.9)	4.97, d (18.1)	5.48, t (7.7)
				5.10, d (18.1)	
3′	1.50, t (6.3)	3.48, m	3.17, t (9.4)		3.49, m
		3.68, dd (4.1, 12.8)	3.47, dd (4.1,12.3)		
4′					
5′			6.96, d (7.1)		7.34, s
6′		7.49, d (6.6)	6.58, d (7.1)		
7′		6.91, t (6.2)			7.41, s
8′		7.03, t (6.1)	6.58, d (7.1)		
9′		7.29, d (6.8)	6.96, d (7.1)		
11′		10.94, s			
12′		7.14, d (1.9)			
4″	3.56, m	3.56, m	3.56, m	3.49, m	
5″	3.81, m	3.62, m	3.61, m	3.62, m	6.65, d (6.8)
6″	1.07, m	0.89, d (5.2)	0.98, d (5.4)	0.92, d (6.2)	1.82, d (6.8)
7″	1.14, ov. *^c^*	0.92, d (5.6)	1.01, d (5.6)	0.96, d (6.7)	1.77, s

*^a^* Measured in DMSO-*d*_6_; *^b^* Measured in CDCl_3_; ^*c*^ ov.: overlapped signal; *^d^* Measured at 500 MHz; *^e^* Measured at 600 MHz.

## Supplementary Materials

The following are available online at https://www.mdpi.com/1660-3397/17/5/253/s1, Figure S1: HPLC analysis of the crude of HDN151398; Figure S2: The 18S rRNA sequences data of HDN151398; Figures S3–S32: 1D and 2D NMR spectra, IR spectra, HRESIMS spectra, and UV spectra of **1**–**3**; Figures S33–S34: HPLC analysis of the FDAA derivatives of the compounds **1**–**3** and l-Me-glutamate and d-Me-glutamate; Table S1: Cytotoxicities of compounds **1**–**5** against twelve cancer cell lines; Figures S35–S44: ^1^H NMR spectra and HRESIMS spectra of **6**–**10**.

## References

[1] Wang Y.T., Xue Y.R., Liu C.H. (2015). A Brief Review of Bioactive Metabolites Derived from Deep-Sea Fungi. Mar. Drugs.

[2] Tortorella E., Tedesco P., Palma Esposito F., January G.G., Fani R., Jaspars M., de Pascale D. (2018). Antibiotics from Deep-Sea Microorganisms: Current Discoveries and Perspectives. Mar. Drugs.

[3] Luo X., Lin X., Tao H., Wang J., Li J., Yang B., Zhou X., Liu Y. (2018). Isochromophilones A-F, Cytotoxic Chloroazaphilones from the Marine Mangrove Endophytic Fungus *Diaporthe* sp. SCSIO 41011. J. Nat. Prod..

[4] Gao J.M., Yang S.X., Qin J.C. (2013). Azaphilones: chemistry and biology. Chem. Rev..

[5] Chen M., Shen N.X., Chen Z.Q., Zhang F.M., Chen Y. (2017). Penicilones A-D, Anti-MRSA Azaphilones from the Marine-Derived Fungus *Penicillium janthinellum* HK1-6. J. Nat. Prod..

[6] Li X., Tian Y., Yang S.X., Zhang Y.M., Qin J.C. (2013). Cytotoxic azaphilone alkaloids from *Chaetomium globosum* TY1. Bioorg. Med. Chem. Lett..

[7] Wang T.H., Lin T.F. (2007). Monascus Rice Products. Adv. Food Nutr. Res..

[8] Nam J.Y., Kim H.K., Kwon J.Y., Han M.Y., Son K.H., Lee U.C., Choi J.D., Kwon B.M. (2000). 8-O-Methylsclerotiorinamine, antagonist of the Grb2-SH2 domain, isolated from *Penicillium multicolor*. J. Nat. Prod..

[9] Clark R.C., Lee S.Y., Searcey M., Boger D.L. (2009). The isolation, total synthesis and structure elucidation of chlorofusin, a natural product inhibitor of the p53–MDM2 protein–protein interaction. Nat. Prod. Rep..

[10] Musso L., Dallavalle S., Merlini L., Bava A., Nasini G., Penco S., Giannini G., Giommarelli C., De Cesare A., Zuco V. (2010). Natural and semisynthetic azaphilones as a new scaffold for Hsp90 inhibitors. Bioorg. Med. Chem..

[11] Bai J., Lu Y., Xu Y.M., Zhang W., Chen M., Lin M., Gunatilaka A.A., Xu Y., Molnar I. (2016). Diversity-Oriented Combinatorial Biosynthesis of Hybrid Polyketide Scaffolds from Azaphilone and Benzenediol Lactone Biosynthons. Org. Lett..

[12] Makrerougras M., Coffinier R., Oger S., Chevalier A., Sabot C., Franck X. (2017). Total Synthesis and Structural Revision of Chaetoviridins, A. Org. Lett..

[13] Takahashi M., Koyama K., Natori S. (1990). Four new azaphilones from *Chaetomium globosum* var. flavo-viridae. Chem. Pharm. Bull..

[14] Wang W., Liao Y., Chen R., Hou Y., Ke W., Zhang B., Gao M., Shao Z., Chen J., Li F. (2018). Chlorinated Azaphilone Pigments with Antimicrobial and Cytotoxic Activities Isolated from the Deep Sea Derived Fungus *Chaetomium* sp. NA-S01-R1. Mar. Drugs.

[15] Steyn P.S., Vleggaar R. (1976). The Structure of Dihydrodeoxy-8-epi-austdiol and the Absolute Configuration of the Azaphilones. J. Chem. Soc. Perkin Transactions.

[16] Yamada T., Muroga Y., Tanaka R. (2009). New azaphilones, seco-chaetomugilins A and D, produced by a marine-fish-derived *Chaetomium globosum*. Mar. Drugs.

[17] Fujii K., Ikai Y., Mayumi T., Oka H., Suzuki M., Harada K.I. (1997). A Nonempirical Method Using LC/MS for Determination of the Absolute Configuration of Constituent Amino Acids in a Peptide:  Elucidation of Limitations of Marfey’s Method and of Its Separation Mechanism. Anal. Chem..

[18] Yamada T., Doi M., Shigeta H., Muroga Y., Hosoe S., Numata A., Tanaka R. (2008). Absolute stereostructures of cytotoxic metabolites, chaetomugilins A–C, produced by a *Chaetomium* species separated from a marine fish. Tetrahedron Lett..

[19] Peng J., Gao H., Zhang X., Wang S., Wu C., Gu Q., Guo P., Zhu T., Li D. (2014). Psychrophilins E–H and Versicotide C, Cyclic Peptides from the Marine-Derived Fungus *Aspergillus versicolor* ZLN-60. J. Nat. Prod..

[20] Zhang Z., He X., Liu C., Che Q., Zhu T., Gu Q., Li D. (2016). Clindanones A and B and cladosporols F and G, polyketides from the deep-sea derived fungus *Cladosporium cladosporioides* HDN14-342. RSC Adv..

