# Supplementary material for: New Glutamine-Containing Azaphilone Alkaloids from Deep-Sea-Derived Fungus Chaetomium globosum HDN151398
Source: Mar Drugs. 2019 Apr 28;17(5):253. doi: 10.3390/md17050253 (PMC6562794; doi:10.3390/md17050253)

Supporting Information for

New Glutamine-Containing Azaphilone Alkaloids from Deep-sea-Derived Fungus *Chaetomium globosum* HDN151398

Chunxiao Sun [1], Xueping Ge [1], Shah Mudassir [1], Luning Zhou [1], Guihong Yu [2], Qian Che [1], Guojian Zhang [1,2], Jixing Peng [3], Qianqun Gu [1], Tianjiao Zhu [1,*] and Dehai Li [1,2,4,*]

[1] Key Laboratory of Marine Drugs, Chinese Ministry of Education, School of Medicine and Pharmacy, Ocean University of China, Qingdao 266003, P. R. China; sunchunxiao93@163.com (C.S.); 15610568273@163.com (X.G.); s84mudassir@gmail.com (S.M.); cheqian064@ouc.edu.cn (Q.C.); zhutj@ouc.edu.cn (T.Z.)

[2] Laboratory for Marine Drugs and Bioproducts of Qingdao National Laboratory for Marine Science and Technology, Qingdao, 266237, P. R. China; Yuguihong1990@126.com (G.Y.); zhangguojian@ouc.edu.cn (G.Z.)

[3] Key Laboratory of Testing and Evaluation for Aquatic Product Safety and Quality, Ministry of Agriculture and Rural Affairs, P. R. China; Yellow Sea Fisheries Research Institute, Chinese Academy of Fishery Sciences, Qingdao 266071, China; pengjixing1987@163.com

[4] Open Studio for Druggability Research of Marine Natural Products, Pilot National Laboratory for Marine Science and Technology, Qingdao, 266237, P. R. China; dehaili@ouc.edu.cn

* Correspondence: dehaili@ouc.edu.cn (D.L.); Tel.: 0086-532-82031619; zhutj@ouc.edu.cn (T.Z.); Tel.: 0086-532-82031632

Table of Contents

Figure S1. HPLC analysis of the crude of HDN151398. 4
Figure S2. The 18S rRNA sequences data of *Chaetomium globosum* HDN151398........ 4
Figure S3. ^{1}H NMR (500 MHz, $CDCl_3$) spectrum of compound **1**. 5
Figure S4. ^{13}C NMR (125 MHz, $CDCl_3$) spectrum of compound **1**. 5
Figure S5. DEPT (125 MHz, $CDCl_3$) spectrum of compound **1**. 6
Figure S6. ^{1}H-^{1}H COSY spectrum of compound **1**. 6
Figure S7. HSQC spectrum of compound **1**. 7
Figure S8. ^{1}H-^{13}C HMBC spectrum of compound **1**. 7
Figure S9. NOESY spectrum of compound **1**. 8
Figure S10. HRESIMS spectrum of compound **1**. 8
Figure S11. IR spectrum of compound **1**........ 9
Figure S12. UV spectrum of compound **1**. 9
Figure S13. ^{1}H NMR (500 MHz, CD_3OD) spectrum of compound **2**. 10
Figure S14. ^{13}C NMR (125 MHz, CD_3OD) spectrum of compound **2**. 10
Figure S15. DEPT (125 MHz, CD_3OD) spectrum of compound **2**. 11
Figure S16. ^{1}H-^{1}H COSY spectrum of compound **2**. 11
Figure S17. HSQC spectrum of compound **2**. 12
Figure S18. ^{1}H-^{13}C HMBC spectrum of compound **2**. 12
Figure S19. NOESY spectrum of compound **2**. 13
Figure S20. HRESIMS spectrum of compound **2**. 13
Figure S21. IR spectrum of compound **2**........ 14
Figure S22. UV spectrum of compound **2**. 14
Figure S23. ^{1}H NMR (500 MHz, $CDCl_3$) spectrum of compound **3**. 15
Figure S24. ^{13}C NMR (125 MHz, $CDCl_3$) spectrum of compound **3**. 15
Figure S25. DEPT (125 MHz, $CDCl_3$) spectrum of compound **3**. 16
Figure S26. ^{1}H-^{1}H COSY spectrum of compound **3**. 16
Figure S27. HSQC spectrum of compound **3**. 17
Figure S28. ^{1}H-^{13}C HMBC spectrum of compound **3**. 17
Figure S29. NOESY spectrum of compound **3**. 18
Figure S30. HRESIMS spectrum of compound **3**. 18
Figure S31. IR spectrum of compound **3**........ 19
Figure S32. UV spectrum of compound **3**. 19
Figure S33. HPLC analysis of the FDAA derivatives of the compounds **1**, **3** and L-Me-glutamate and D-Me-glutamate. 20
Figure S34. HPLC analysis of the FDAA derivatives of the compound **2** and L-glutamate and D-glutamate. 20
Table S1. Cytotoxicities of compounds **1-5** against twelve cancer cell Lines. 21
Figure S35. ^{1}H NMR (500 MHz, $CDCl_3$) spectrum of compound **6**. 21
Figure S36. HRESIMS spectrum of compound **6**. 22
Figure S37. ^{1}H NMR (600 MHz, DMSO-d_6) spectrum of compound **7**. 22
Figure S38. HRESIMS spectrum of compound **7**. 23
Figure S39. ^{1}H NMR (600 MHz, DMSO-d_6) spectrum of compound **8**. 23
Figure S40. HRESIMS spectrum of compound **8**. 24

Figure S41. ^{1}H NMR (500 MHz, DMSO-d_6) spectrum of compound **9**. 24
Figure S42. HRESIMS spectrum of compound **9**. 25
Figure S43. ^{1}H NMR (600 MHz, DMSO-d_6) spectrum of compound **10**. 25
Figure S44. HRESIMS spectrum of compound **10**. 26

Figure S1. HPLC analysis of the crude of HDN151398.

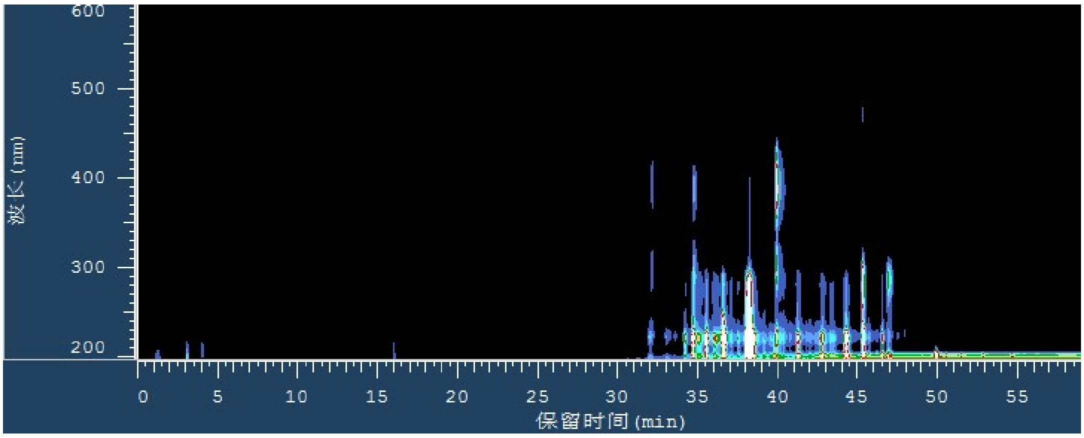

Figure S2. The 18S rRNA sequences data of *Chaetomium globosum* HDN151398.

AAGGAGCTGCAACTCCCTAACCATTGTGAACGTTACCTATACCGTTGCTTC
GGCGGGCGGCCCCGGGGTTTACCCCCCGGGCGCCCCTGGGCCCCACCGCG
GGCGCCCGCCGGAGGTCACCAAACTCTTGATAATTTATGGCCTCTCTGAGT
CTTCTGTACTGAATAAGTCAAAACTTTCAACAACGGATCTCTTGGTTCTGG
CATCGATGAAGAACGCAGCGAAATGCGATAAGTAATGTGAATTGCAGAATT
CAGTGAATCATCGAATCTTTGAACGCACATTGCGCCCGCCAGCATTCTGGC
GGGCATGCCTGTTCGAGCGTCATTTCAACCATCAAGCCCCCGGGCTTGTGT
TGGGGACCTGCGGCTGCCGCAGGCCCTGAAAAGCAGTGGCGGGCTCGCTG
TCGCACCGAGCGTAGTAGCATACATCTCGCTCTGGTCGCGCCGCGGGTTCC
GGCCGTTAAACCACCTTTTAACCCAAGGTTGACCTCGGATCAGGTAGGAAG
ACCCGCTGAACTTAAGCATATCAAAGCCGGGAAGA

Figure S3. ^{1}H NMR (500 MHz, $CDCl_3$) spectrum of compound **1**.

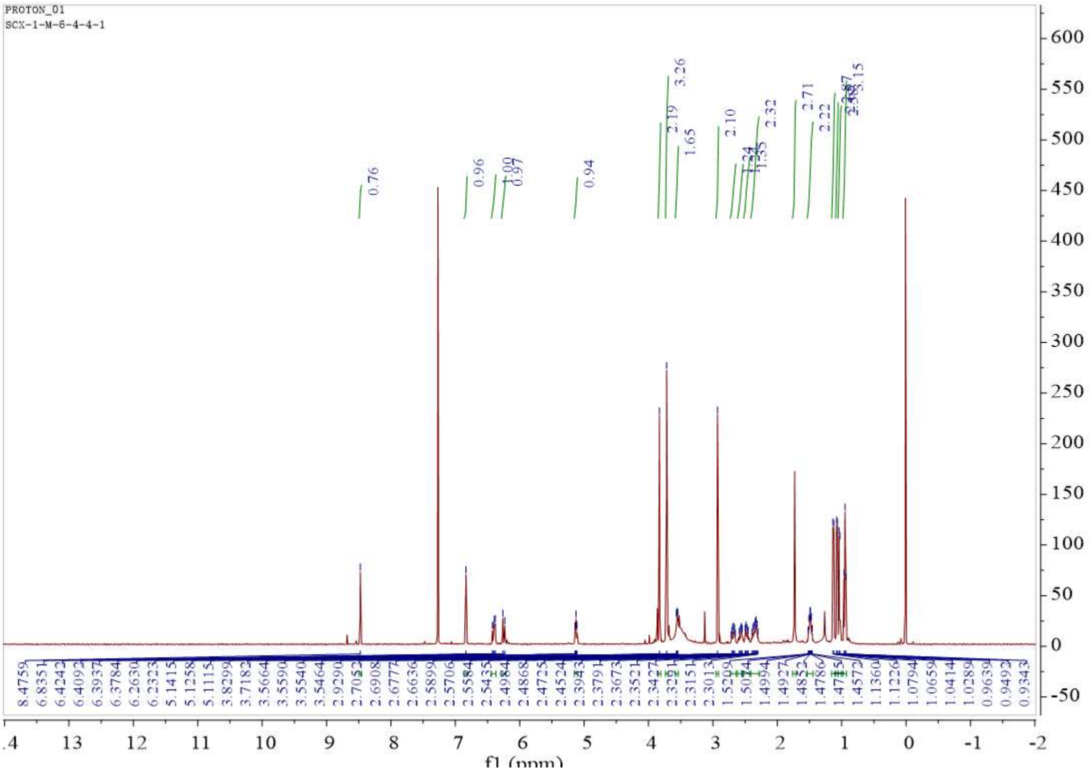

Figure S4. ^{13}C NMR (125 MHz, $CDCl_3$) spectrum of compound **1**.

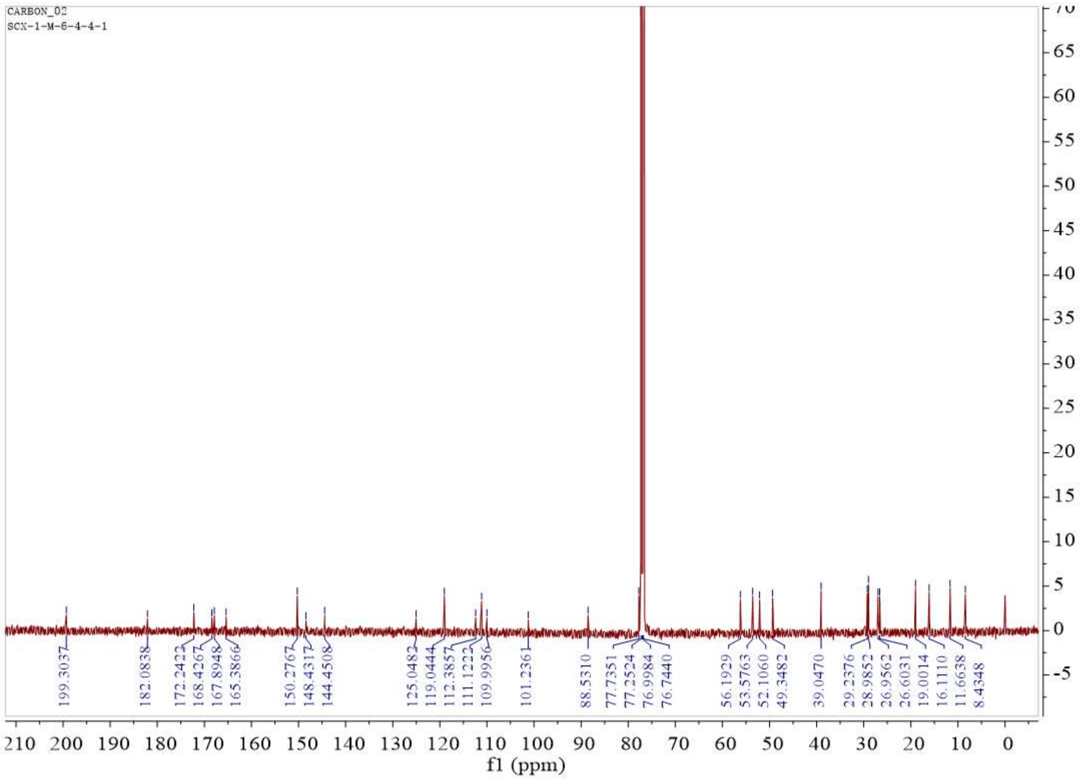

Figure S5. DEPT (125 MHz, $CDCl_3$) spectrum of compound **1**.

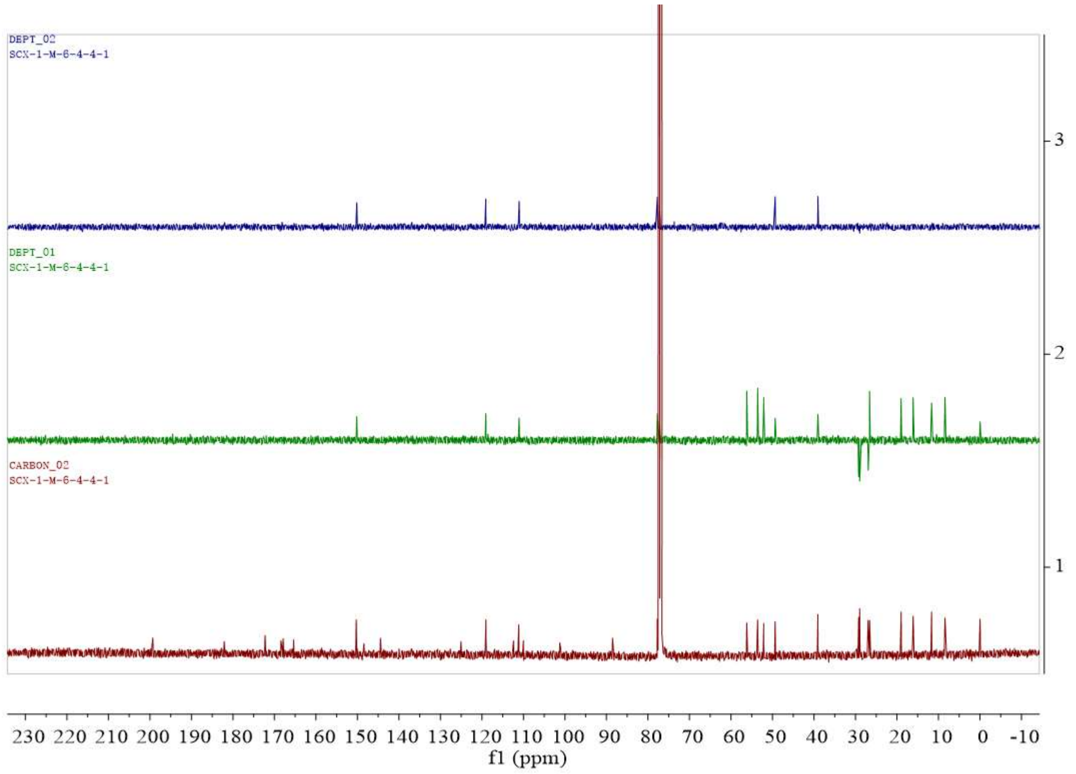

Figure S6. ^{1}H-^{1}H COSY spectrum of compound **1**.

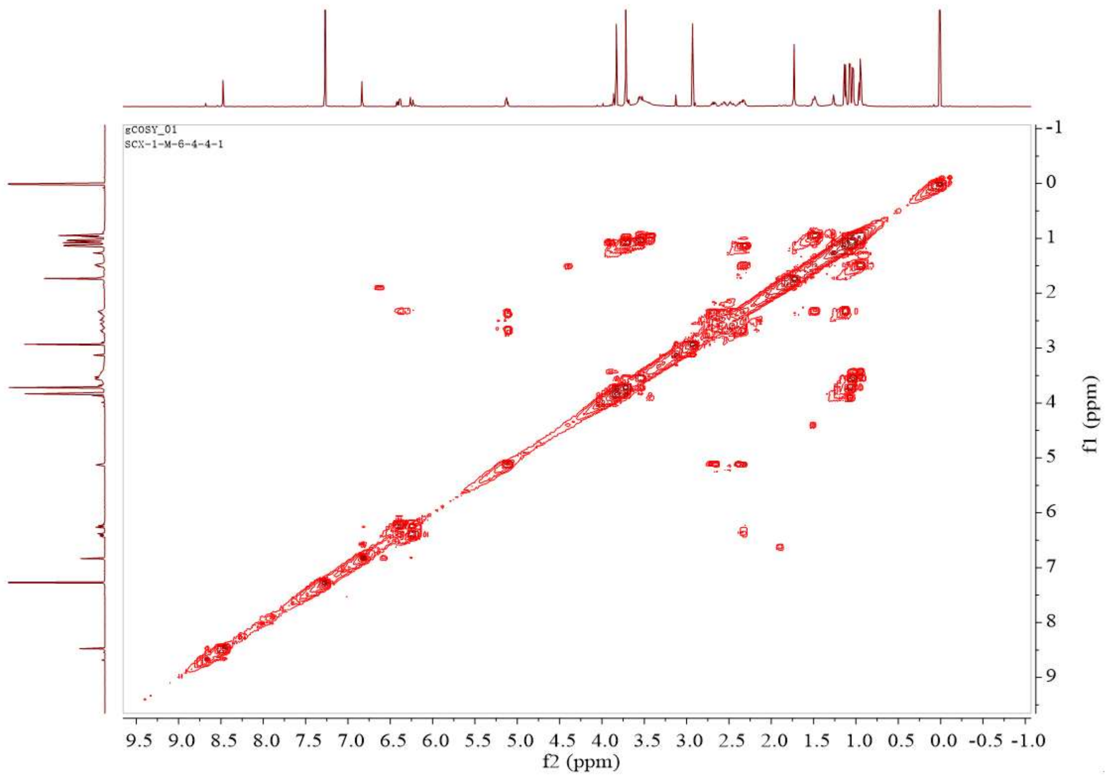

Figure S7. HSQC spectrum of compound **1**.

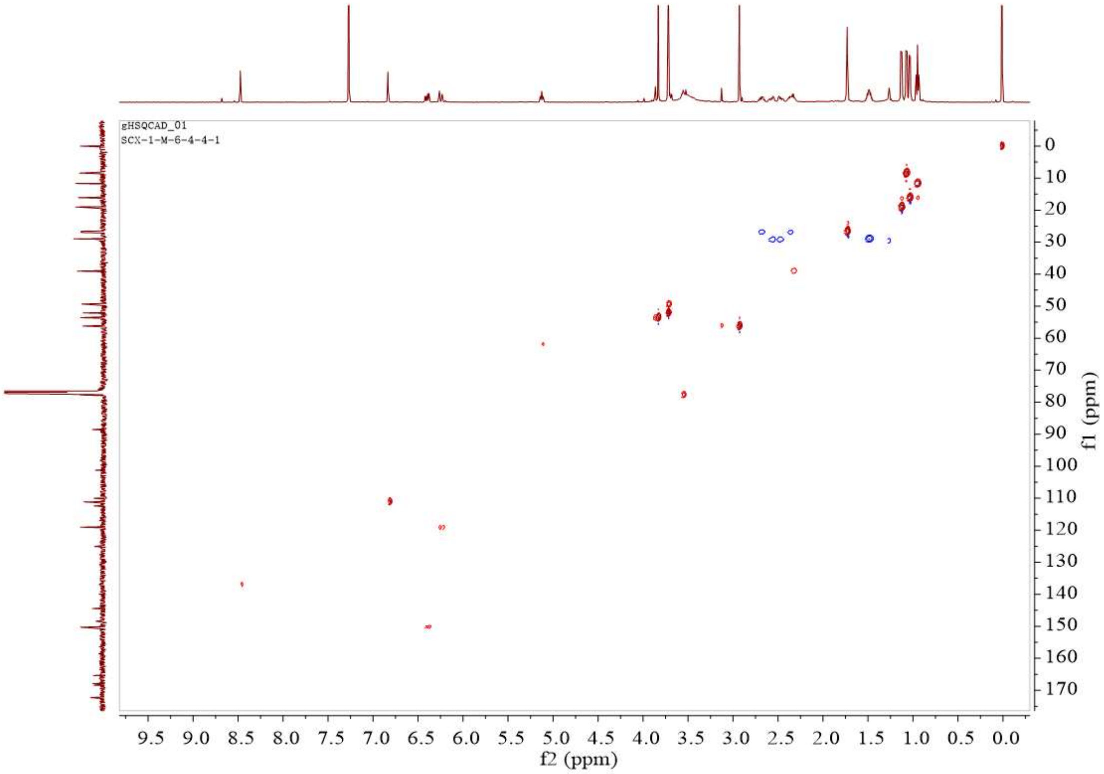

Figure S8. ^{1}H-^{13}C HMBC spectrum of compound **1**.

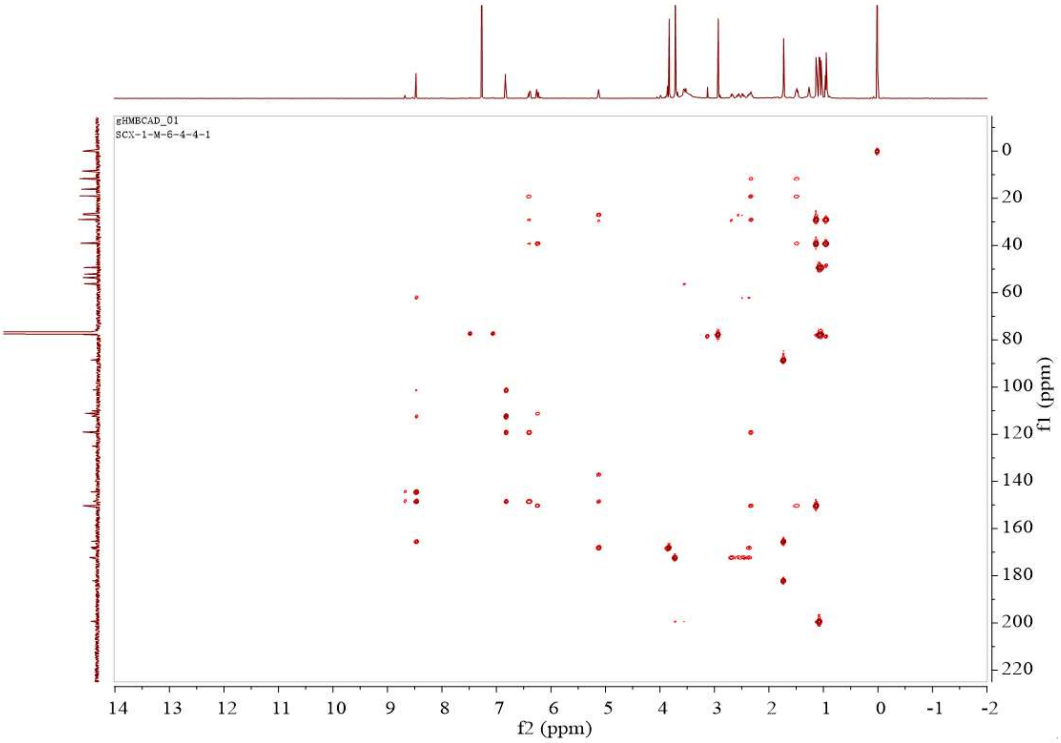

Figure S9. NOESY spectrum of compound **1**.

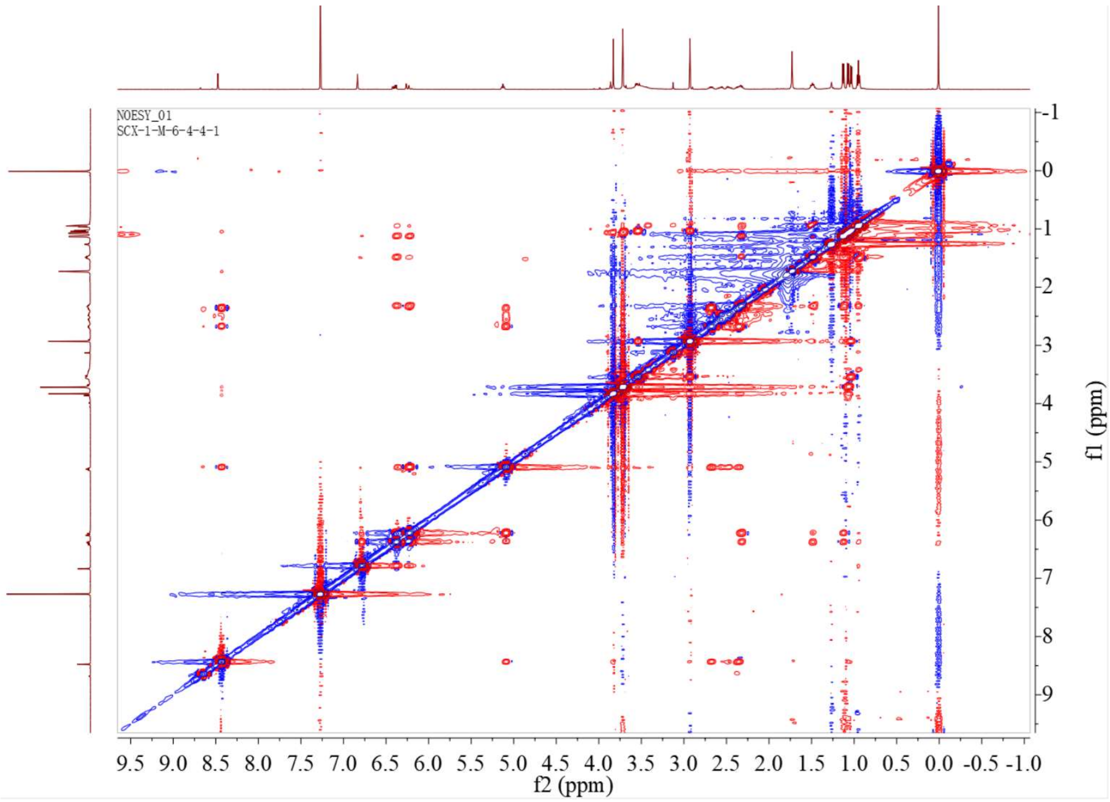

Figure S10. HRESIMS spectrum of compound **1**.

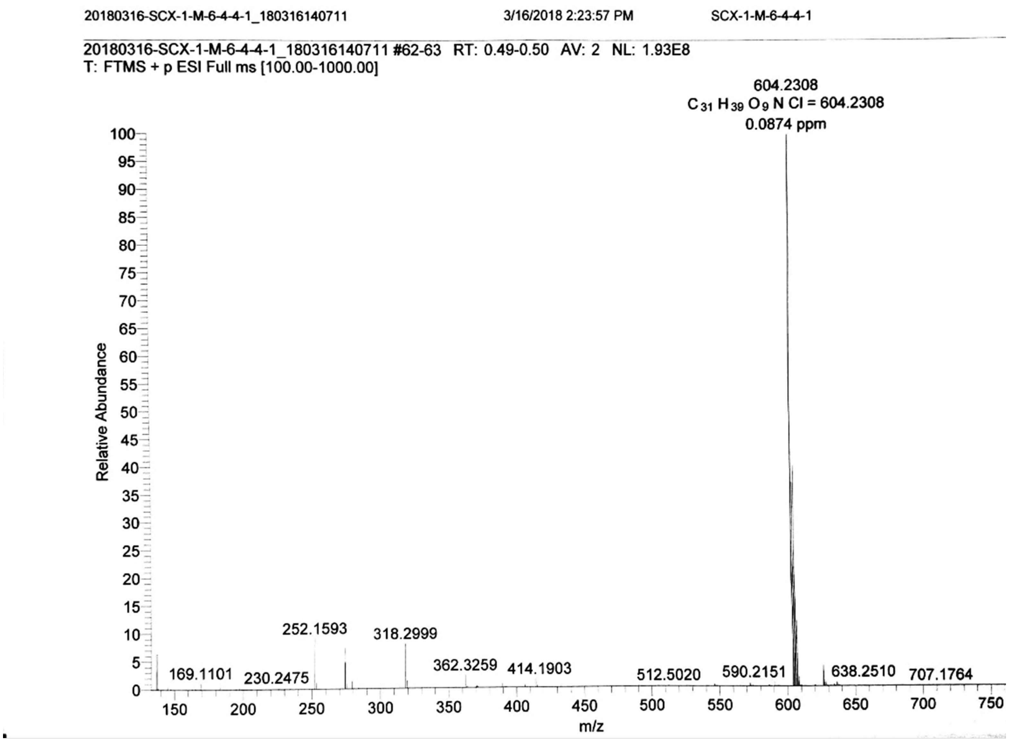

Figure S11. IR spectrum of compound **1**.

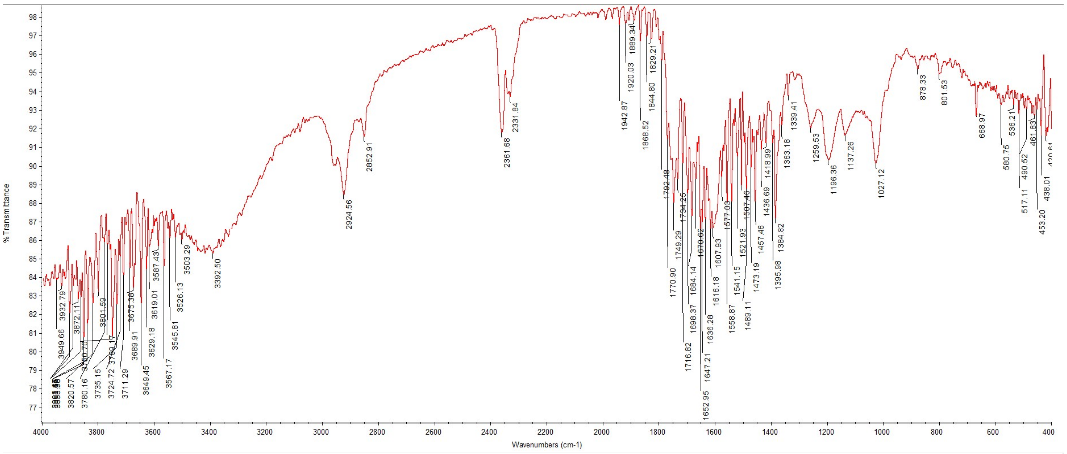

Figure S12. UV spectrum of compound **1**.

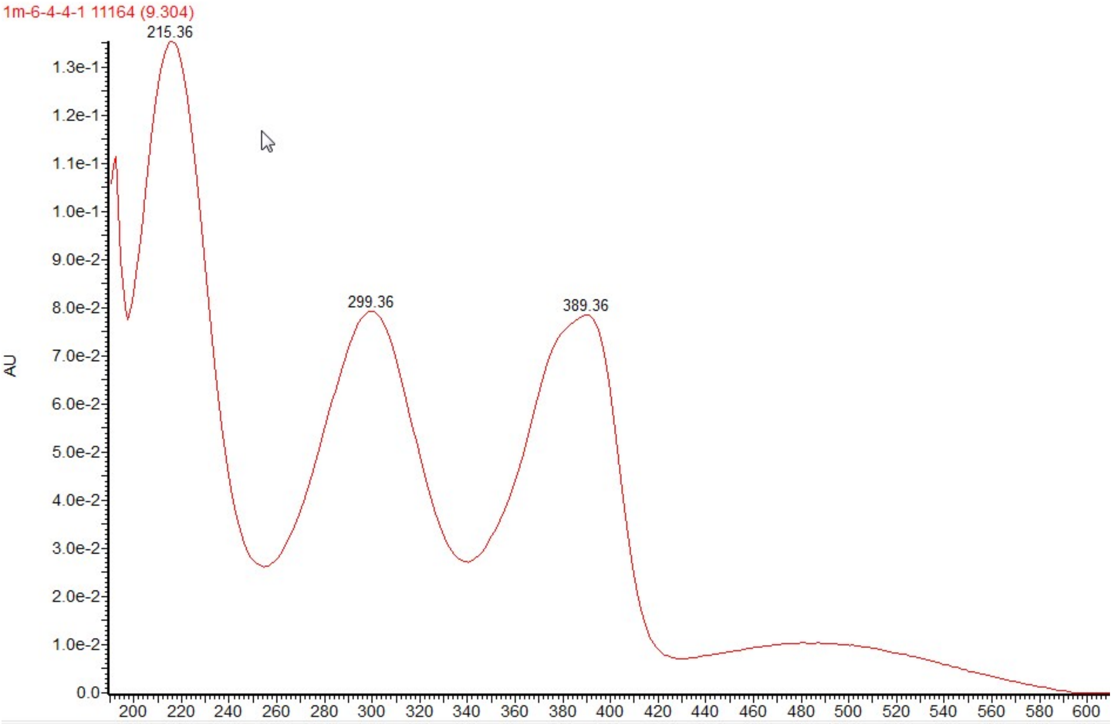

Figure S13. ¹H NMR (500 MHz, CD_3OD) spectrum of compound **2**.

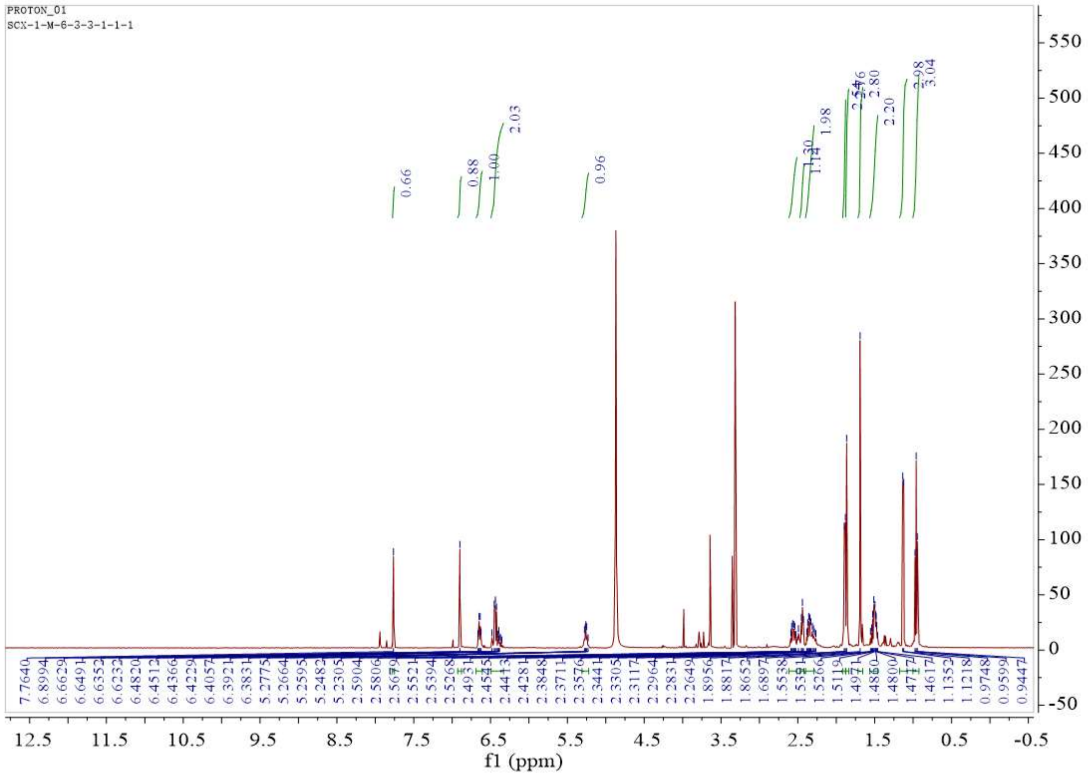

Figure S14. ¹³C NMR (125 MHz, CD_3OD) spectrum of compound **2**.

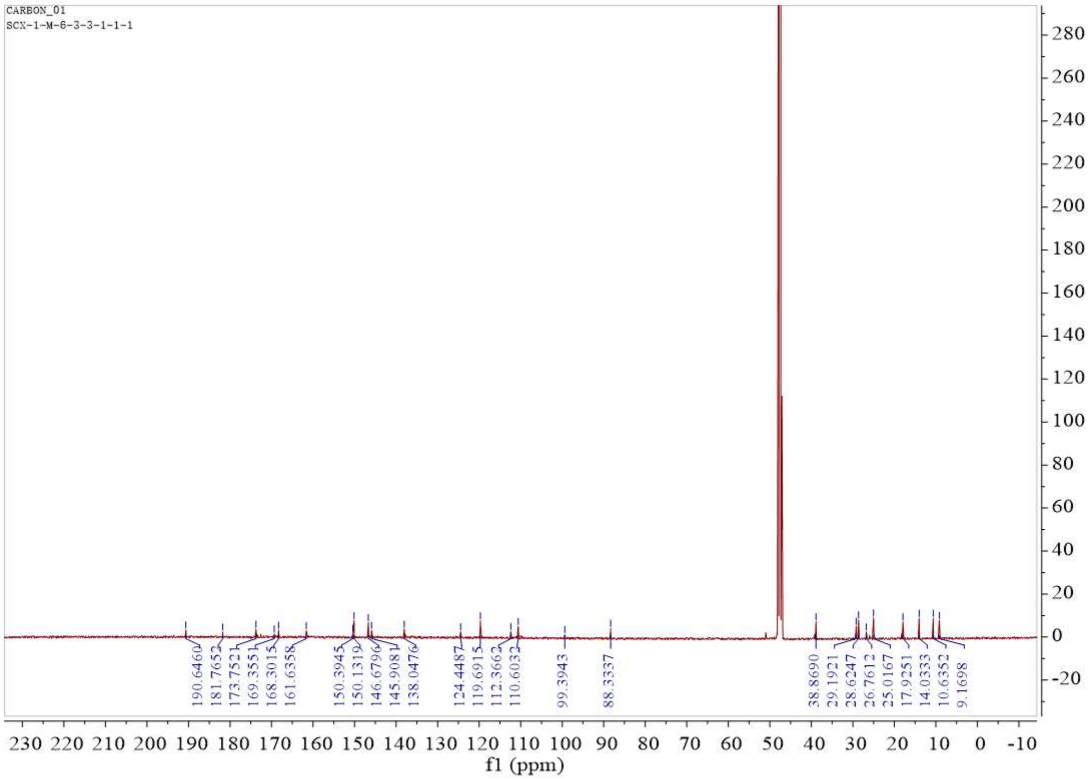

Figure S15. DEPT (125 MHz, CD_3OD) spectrum of compound **2**.

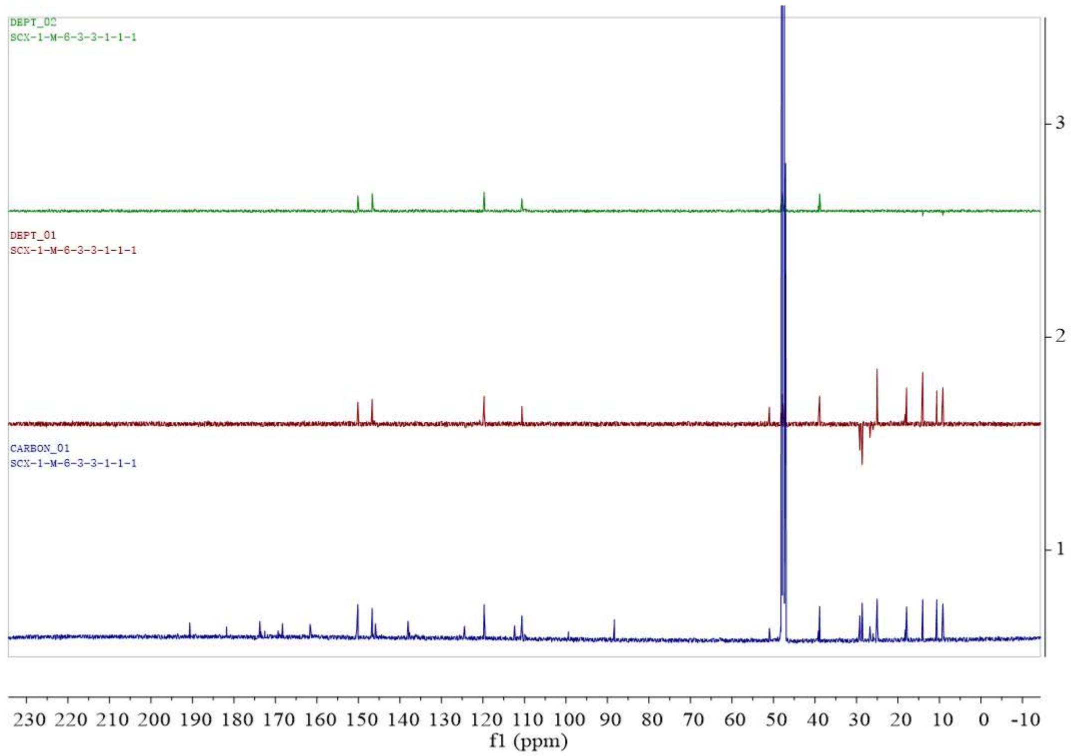

Figure S16. ^{1}H-^{1}H COSY spectrum of compound **2**.

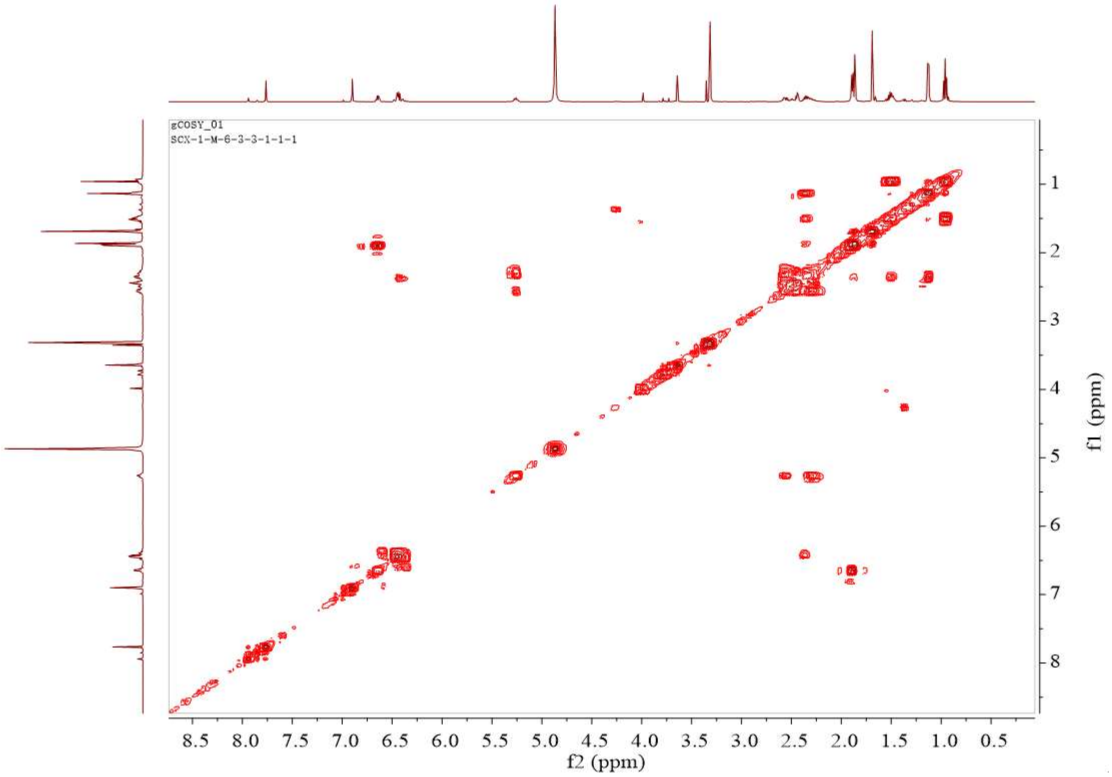

Figure S17. HSQC spectrum of compound **2**.

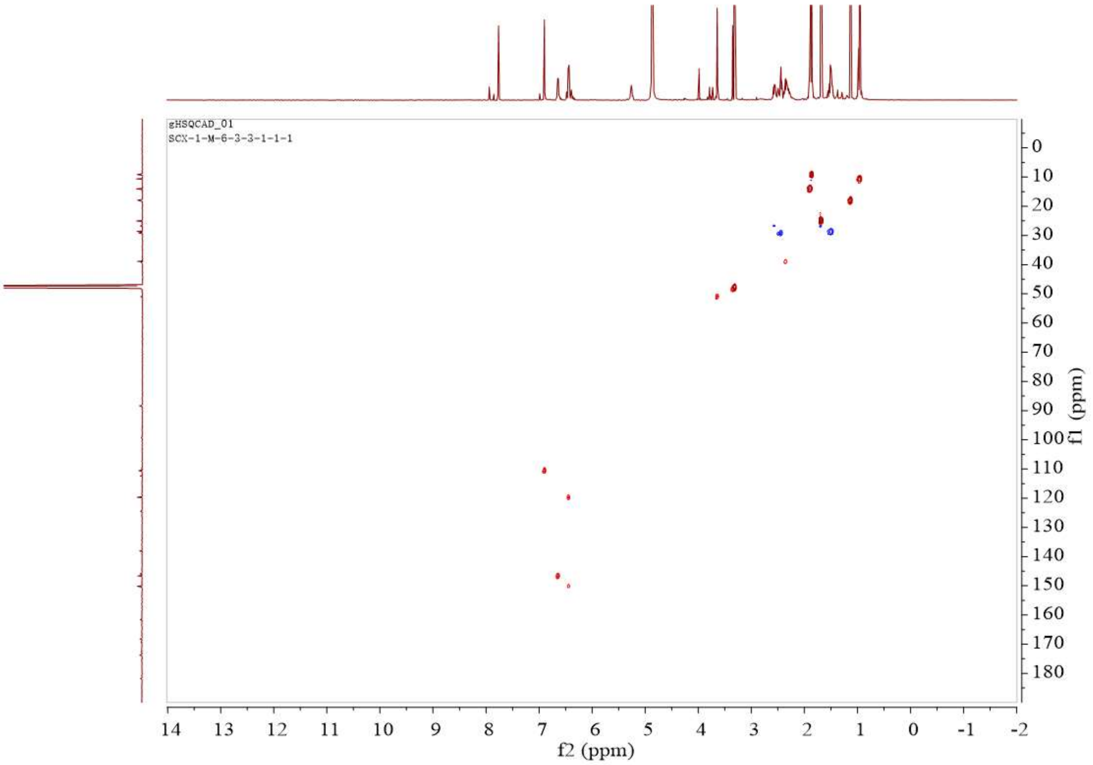

Figure S18. ^{1}H-^{13}C HMBC spectrum of compound **2**.

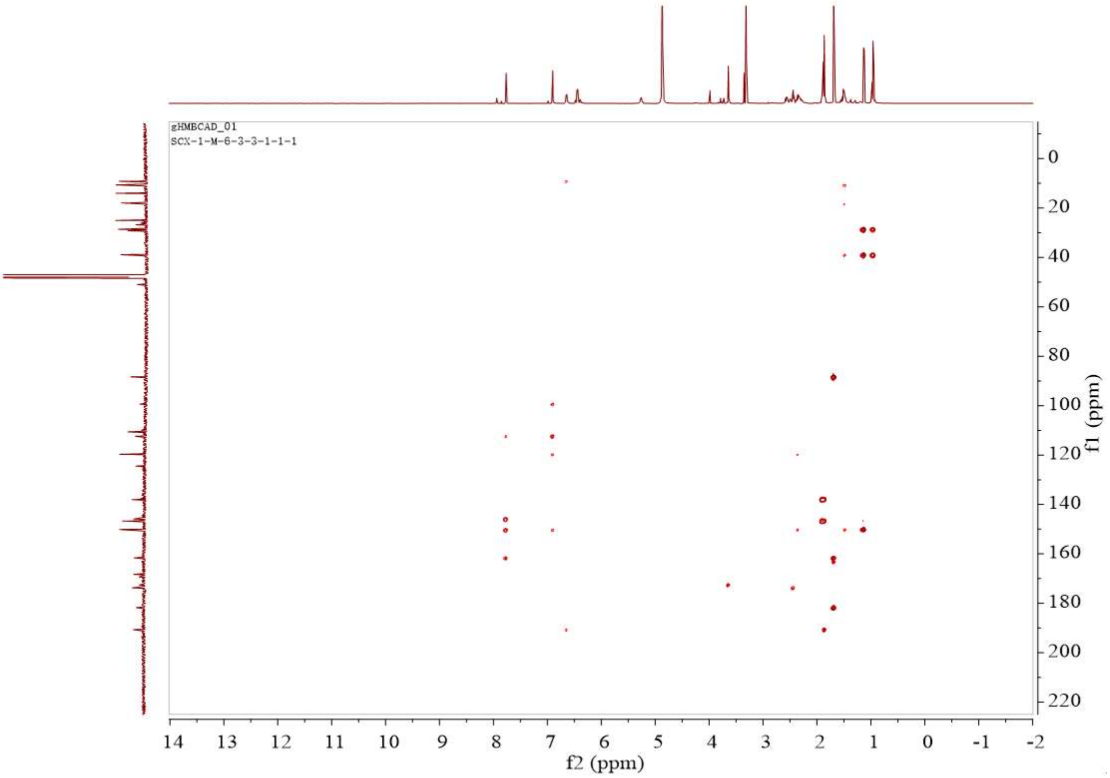

Figure S19. NOESY spectrum of compound **2**.

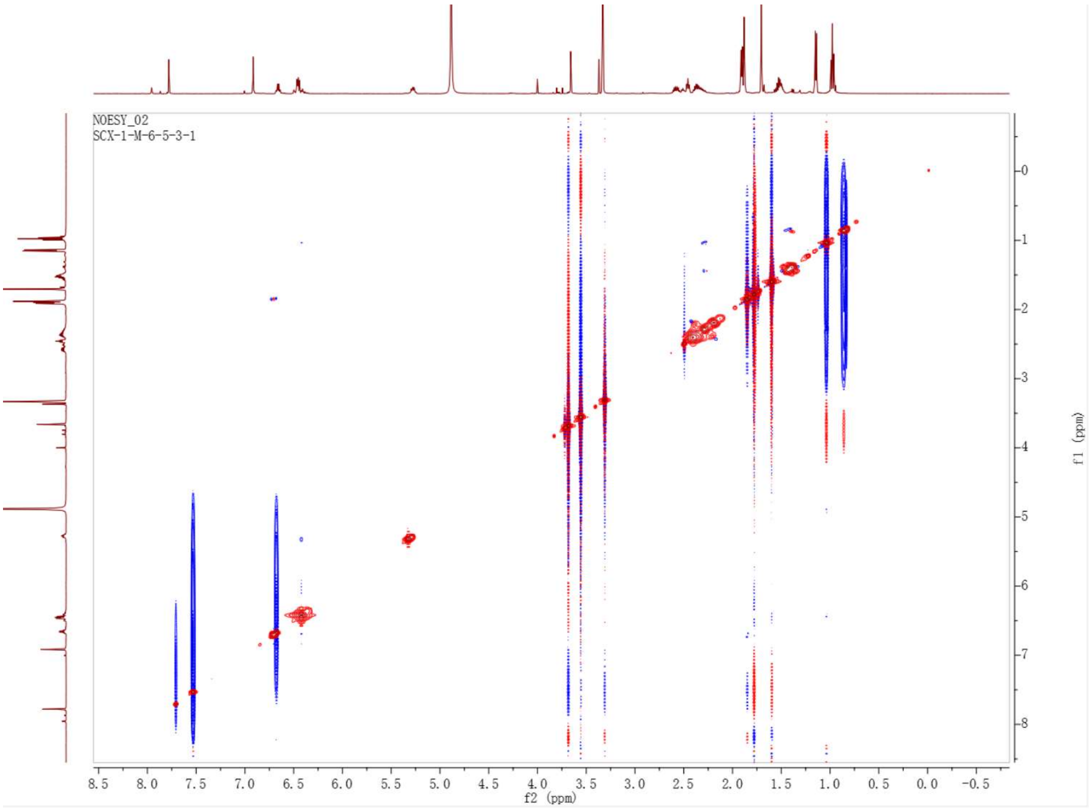

Figure S20. HRESIMS spectrum of compound **2**.

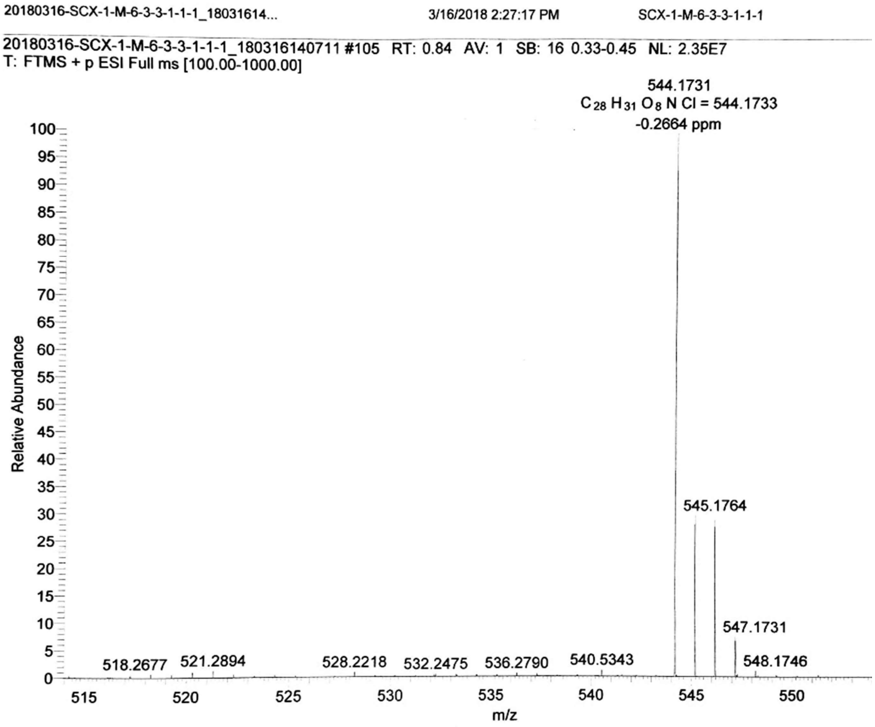

Figure S21. IR spectrum of compound **2**.

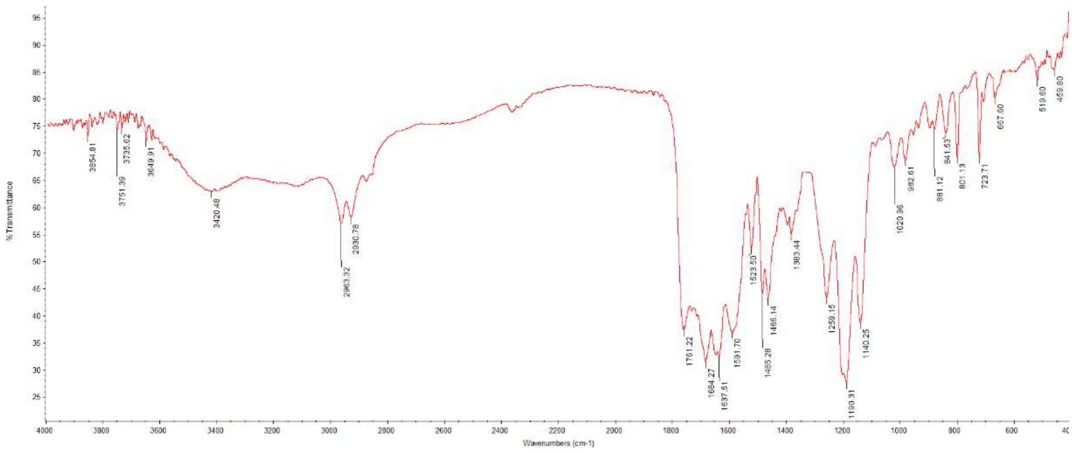

Figure S22. UV spectrum of compound **2**.

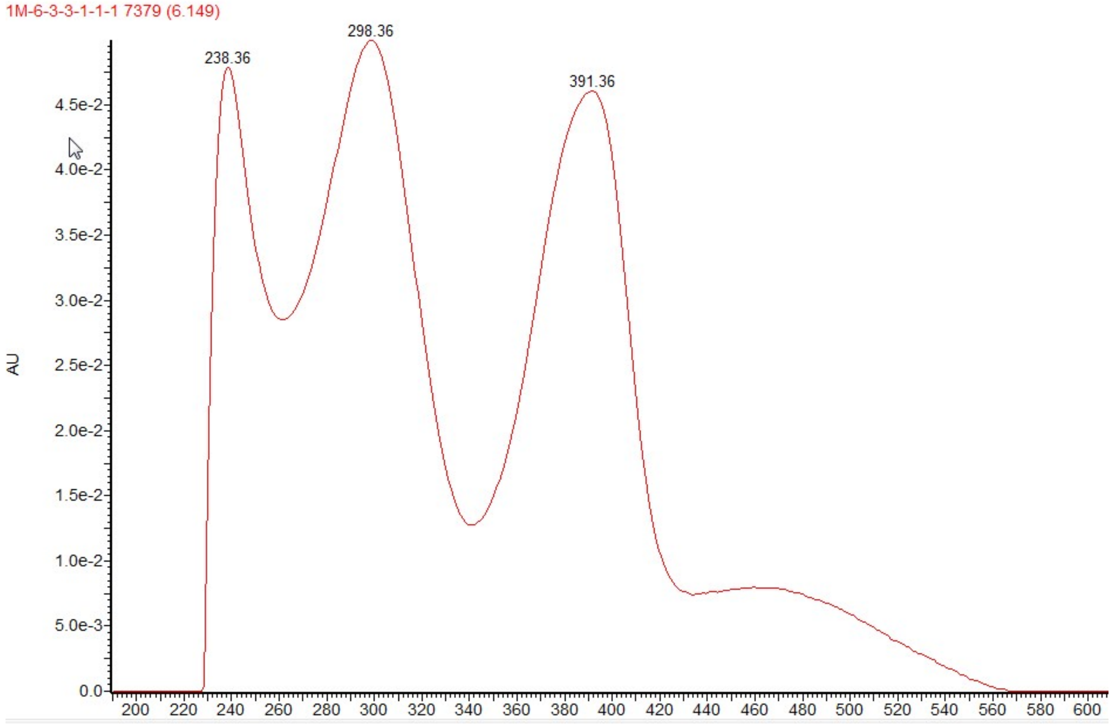

Figure S23. ^{1}H NMR (500 MHz, $CDCl_3$) spectrum of compound **3**.

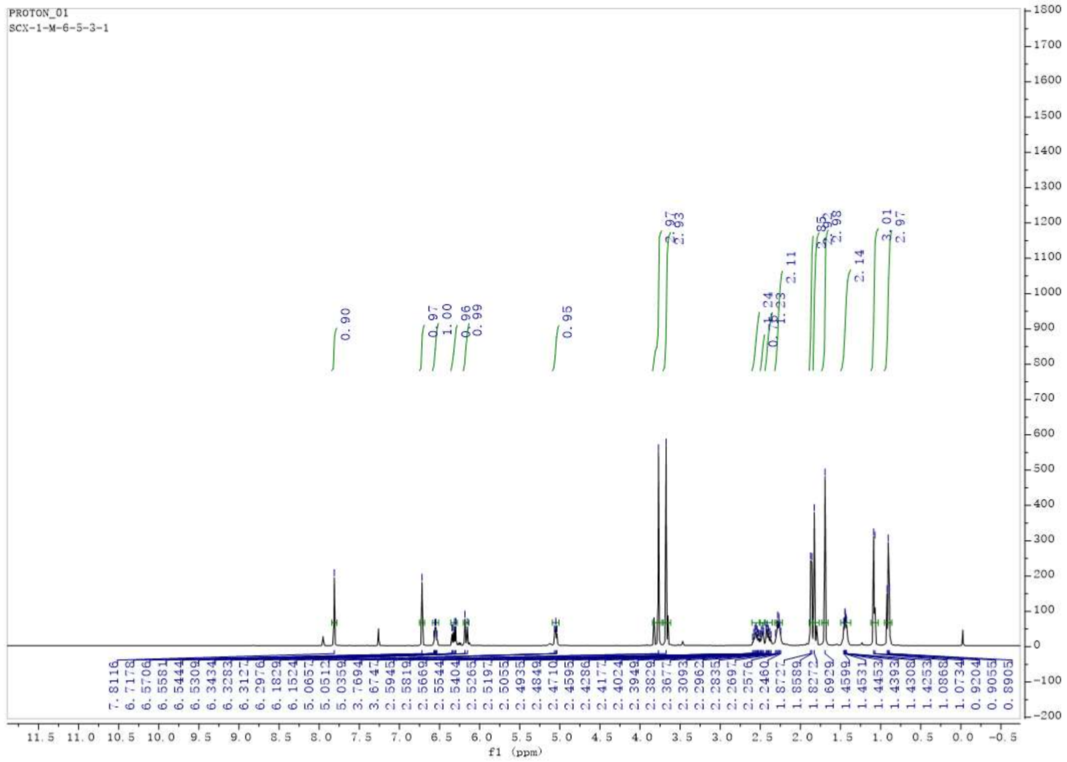

Figure S24. ^{13}C NMR (125 MHz, $CDCl_3$) spectrum of compound **3**.

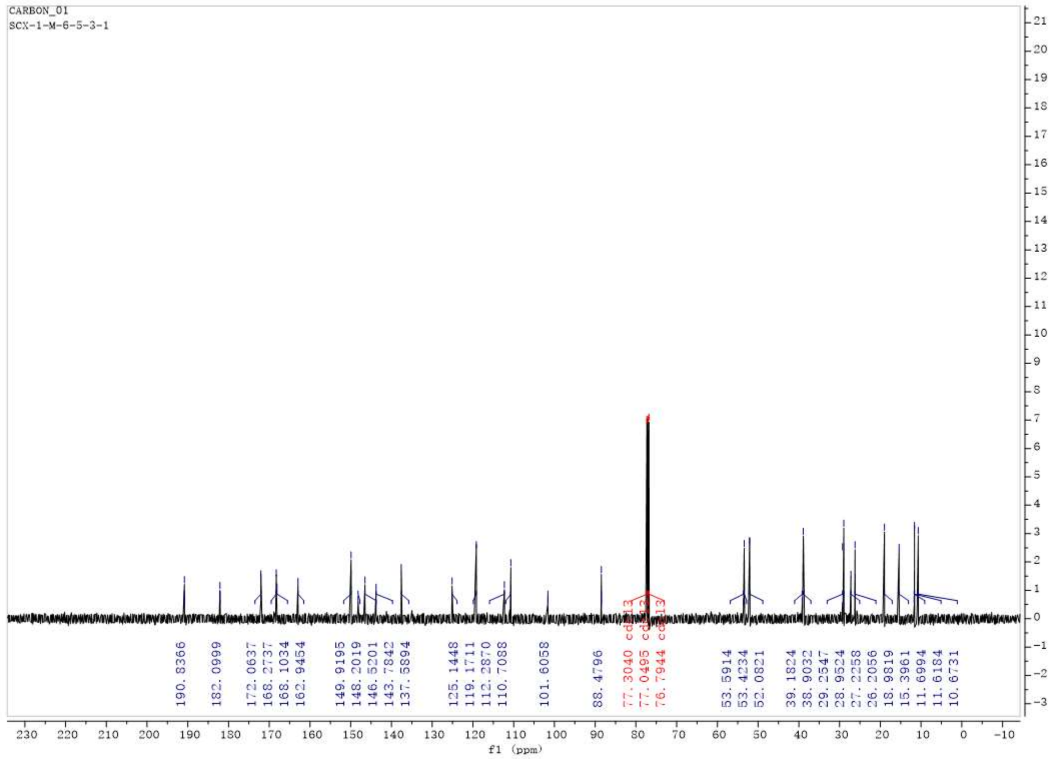

Figure S25. DEPT (125 MHz, $CDCl_3$) spectrum of compound **3**.

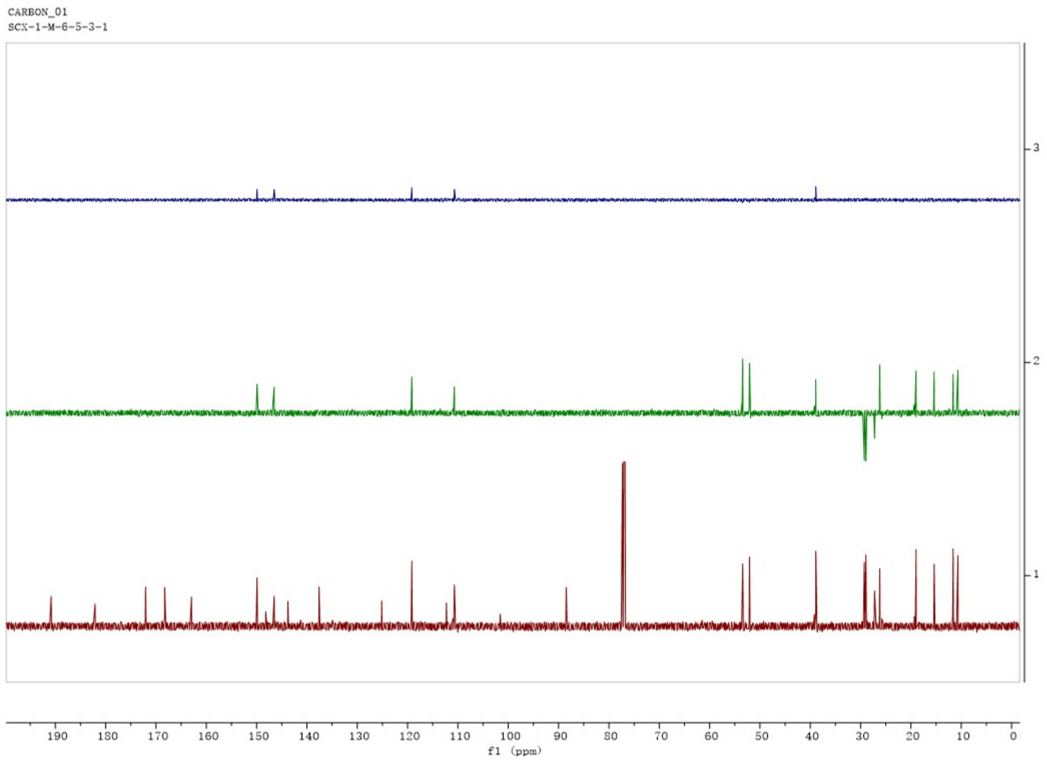

Figure S26. ^1H-^1H COSY spectrum of compound **3**.

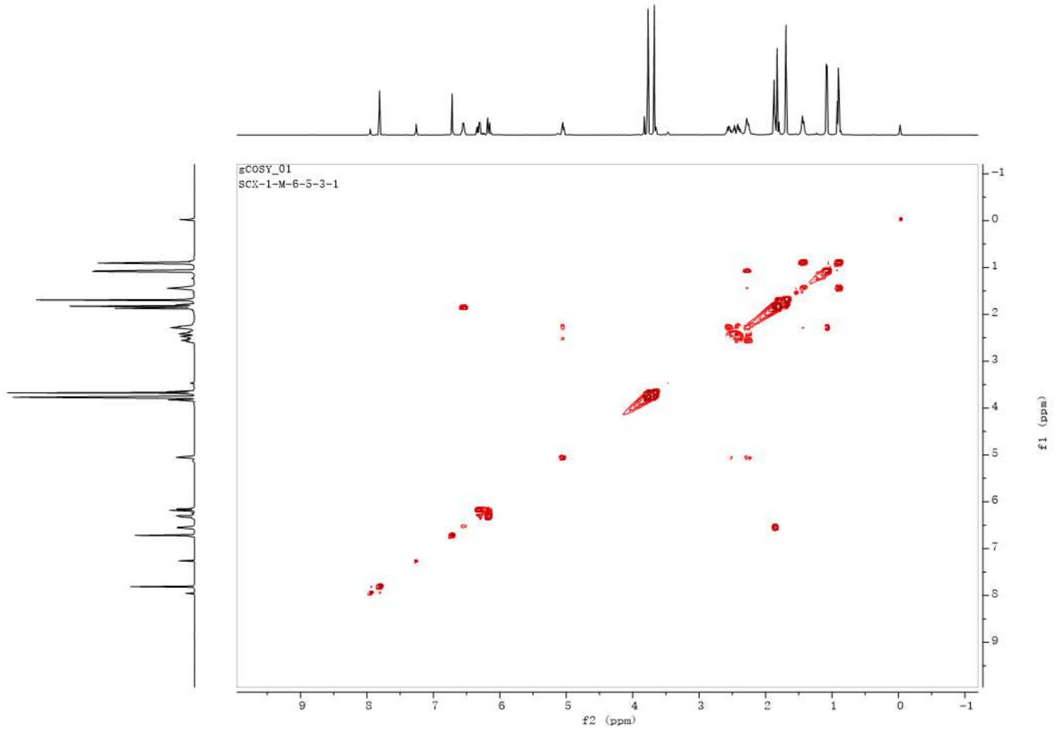

Figure S27. HSQC spectrum of compound **3**.

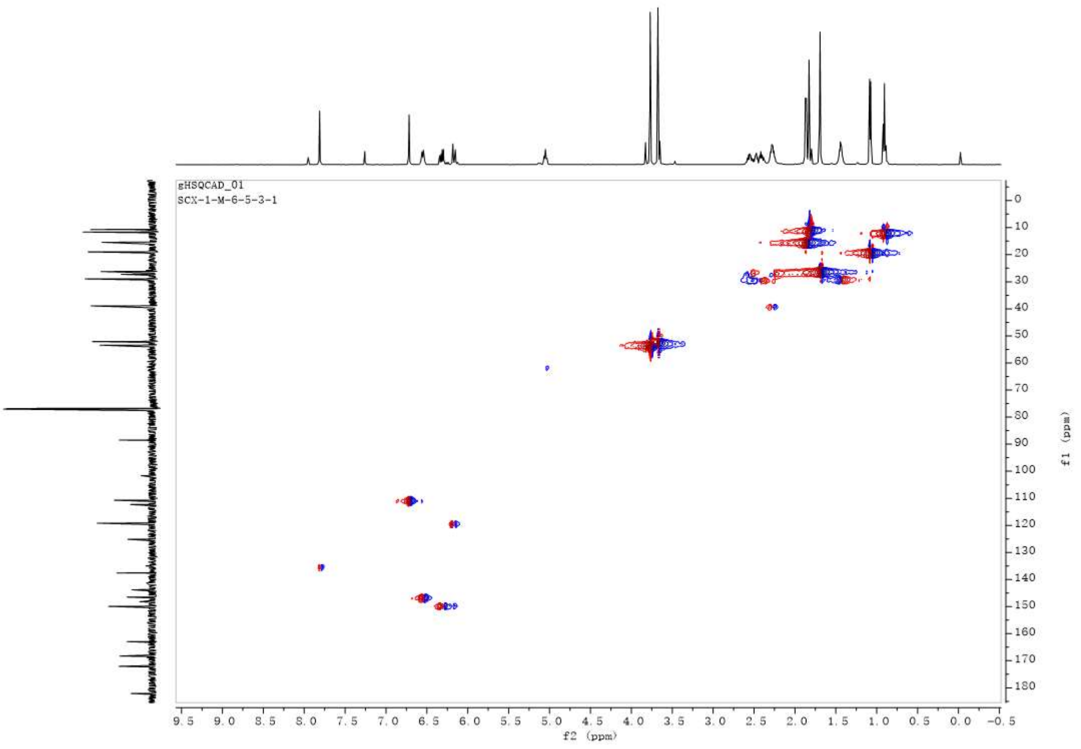

Figure S28. ^{1}H-^{13}C HMBC spectrum of compound **3**.

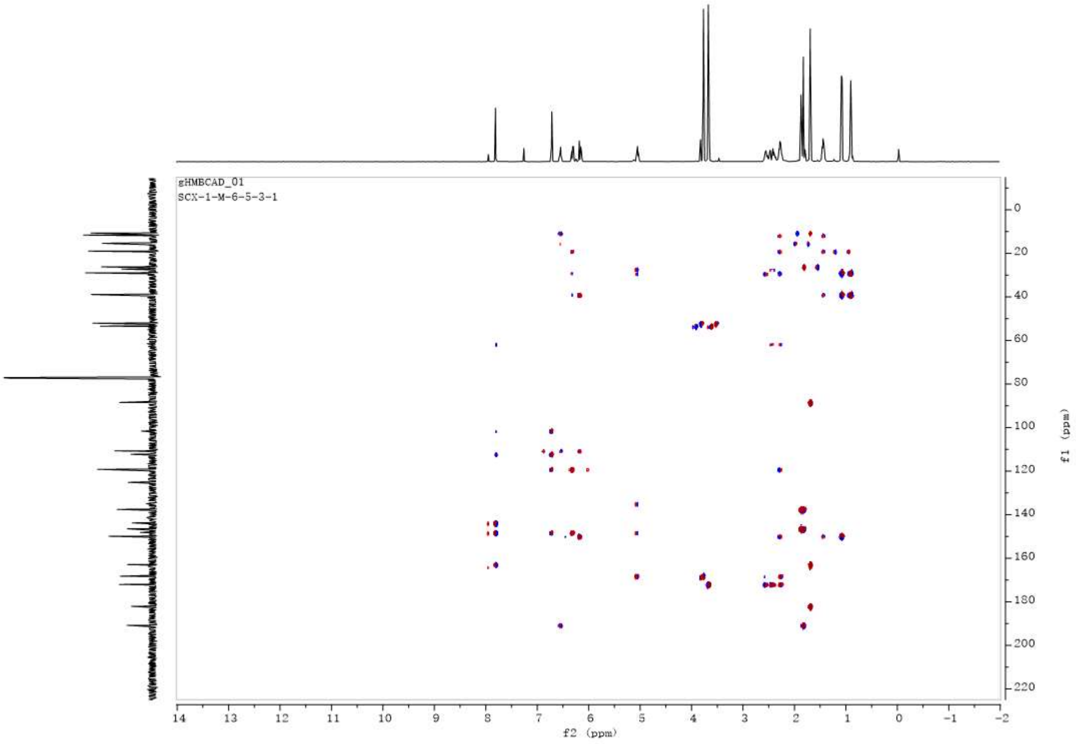

Figure S29. NOESY spectrum of compound **3**.

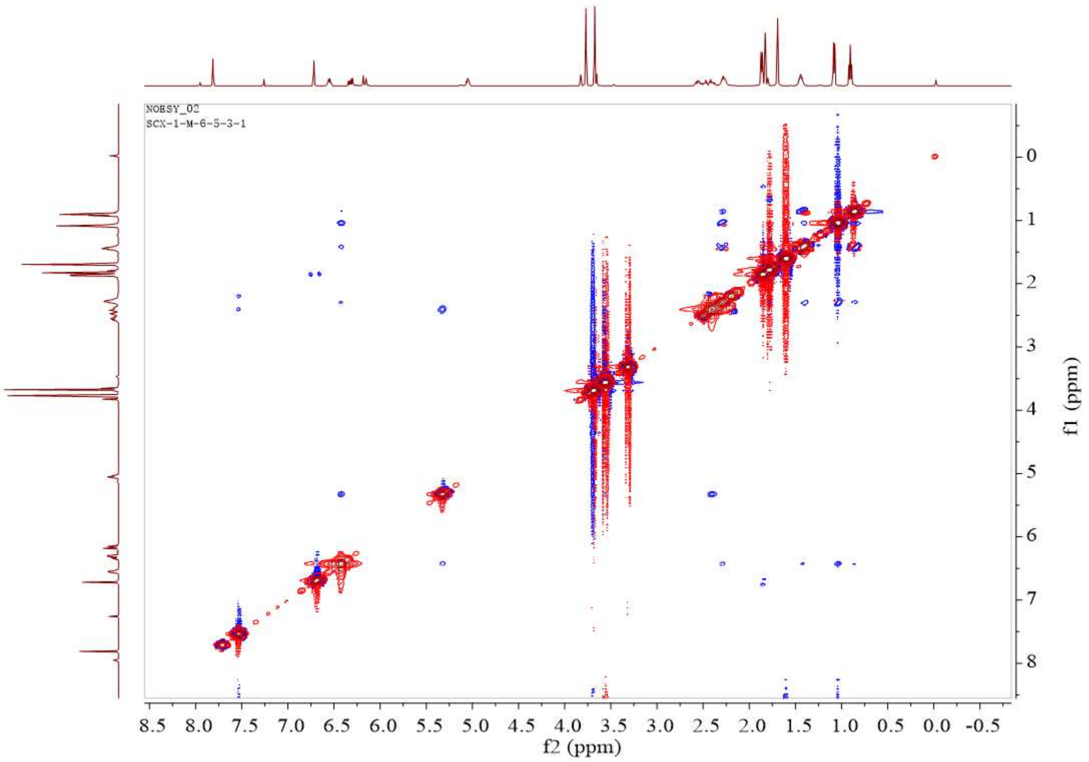

Figure S30. HRESIMS spectrum of compound **3**.

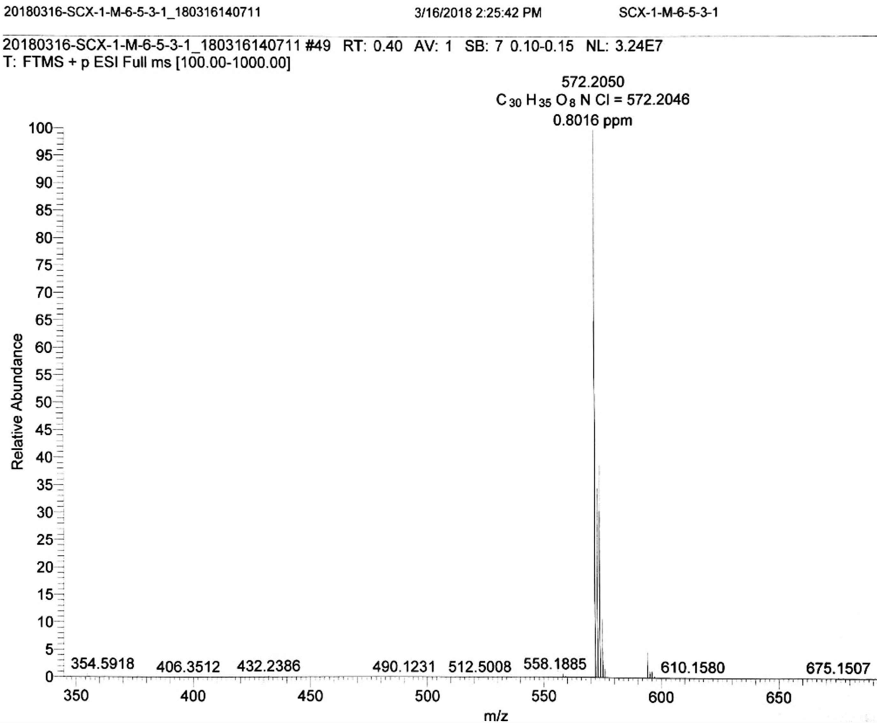

Figure S31. IR spectrum of compound **3**.

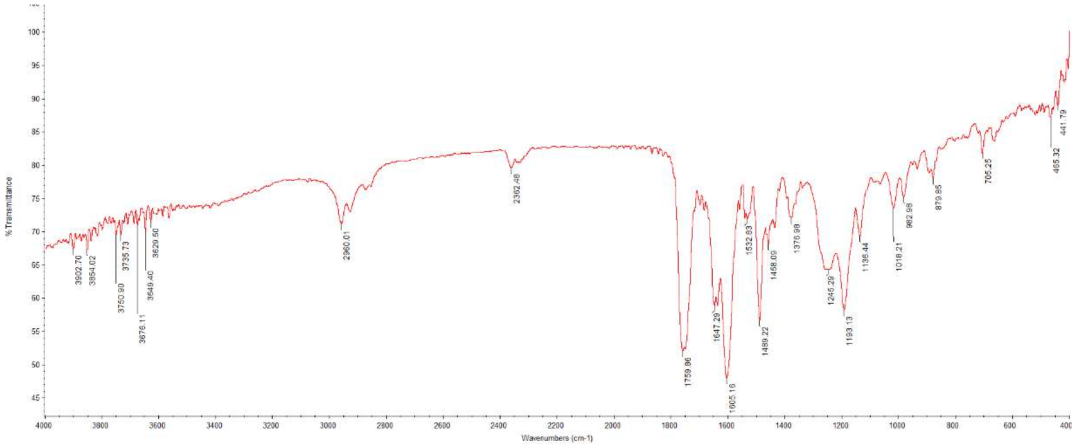

Figure S32. UV spectrum of compound **3**.

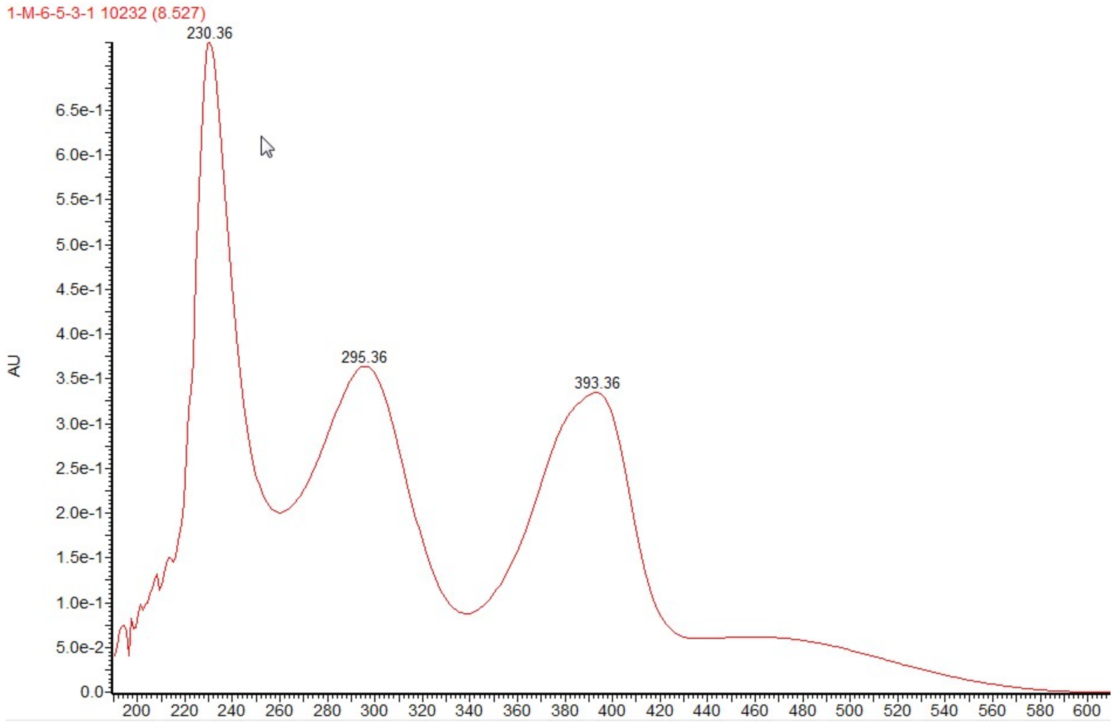

Figure S33. HPLC analysis of the FDAA derivatives of the compounds **1**, **3** and L-Me-glutamate and D-Me-glutamate.

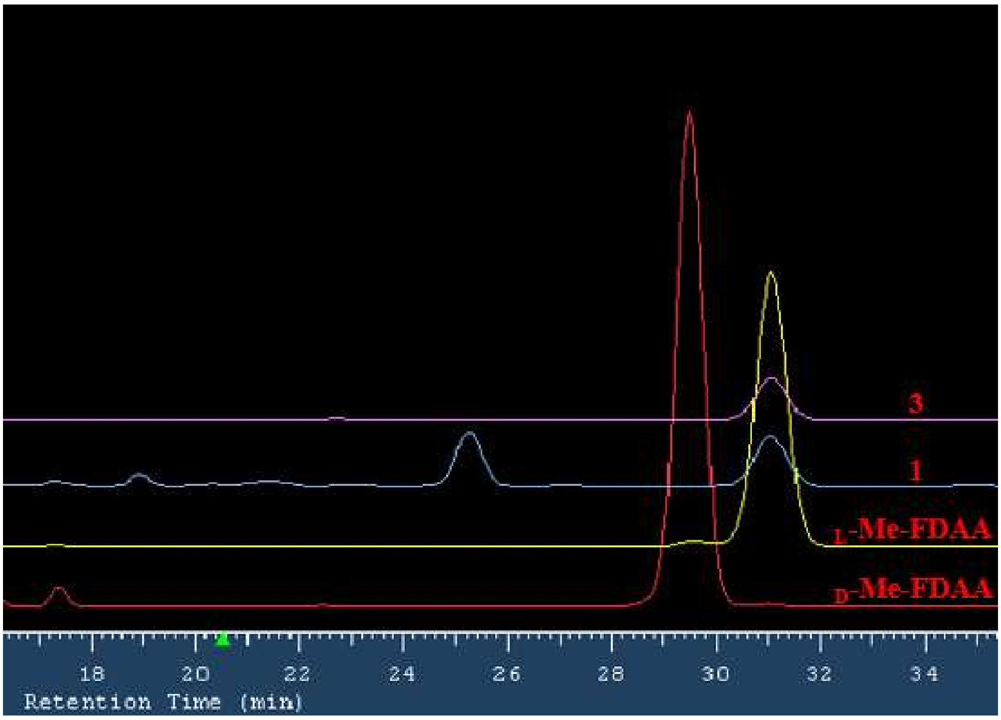

Figure S34. HPLC analysis of the FDAA derivatives of the compound **2** and L-glutamate and D-glutamate.

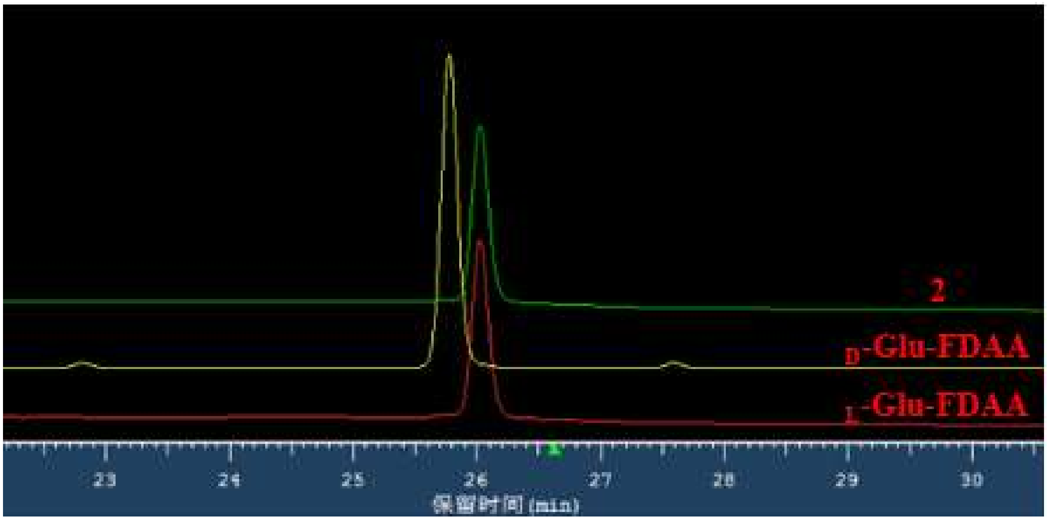

Table S1. Cytotoxicities of compounds **1-5** against twelve cancer cell Lines.

Compounds	IC50 (μM)											
	HL-60	K562	BEL-7402	HCT-116	HeLa	L-02	MGC-803	HO8910	SH-SY5Y	NCl-H1975	U87	MDA-MB-231
**1**	10.3	20.3	23.9	>50	>50	>50	>50	>50	>50	>50	>50	>50
**2**	>50	>50	>50	>50	>50	>50	>50	>50	>50	>50	>50	>50
**3**	11.1	11.7	10.9	11.3	22.1	18.2	6.6	9.7	26.5	11.2	18.3	13.2
**4**	6.4	11.1	17.9	6.1	20.3	15.2	15.3	12.1	23.4	18.3	27.1	22.7
**5**	6.6	12.3	16.8	5.7	13.2	9.1	9.6	8.8	19.4	12.1	17.6	26.6
**ADM**	0.1	0.3	0.4	0.2	0.6	0.4	0.2	0.4	0.2	0.3	0.1	0.2

Figure S35. ^{1}H NMR (500 MHz, $CDCl_3$) spectrum of compound **6**.

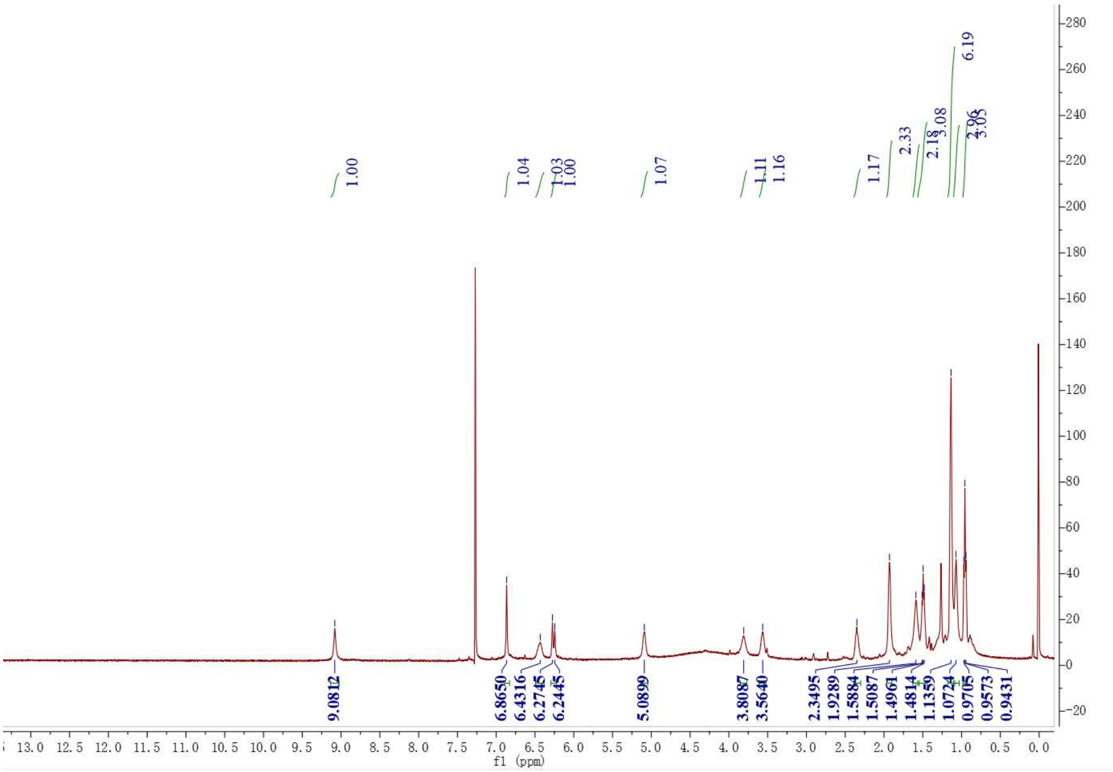

Figure S36. HRESIMS spectrum of compound **6**.

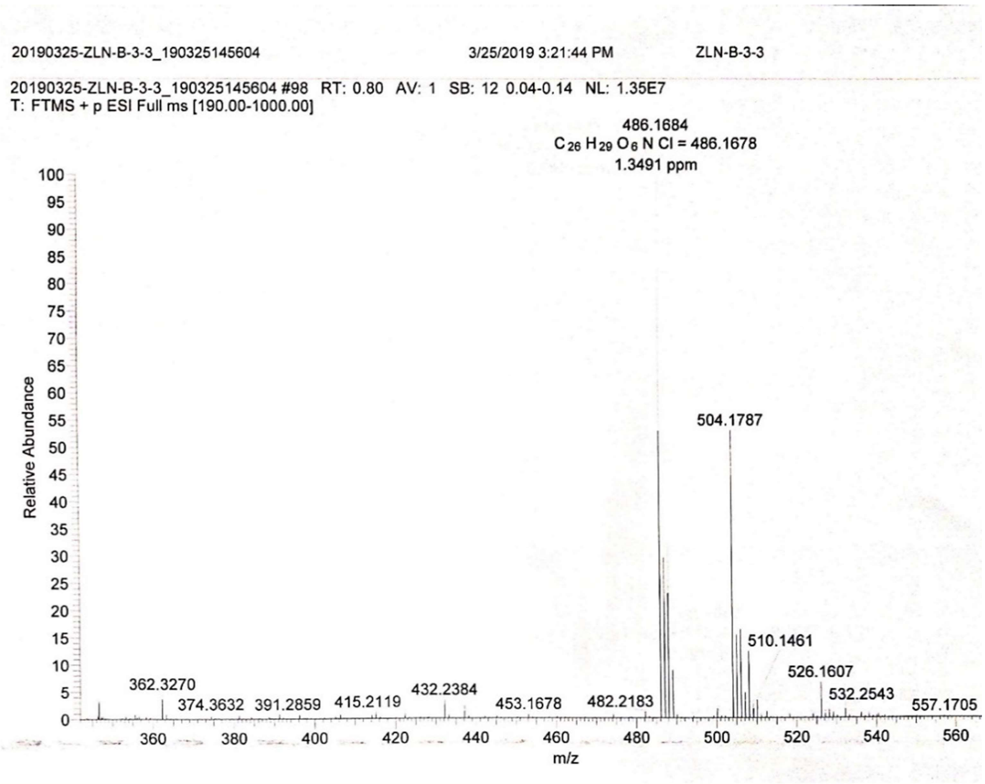

Figure S37. ^{1}H NMR (600 MHz, DMSO-d_6) spectrum of compound **7**.

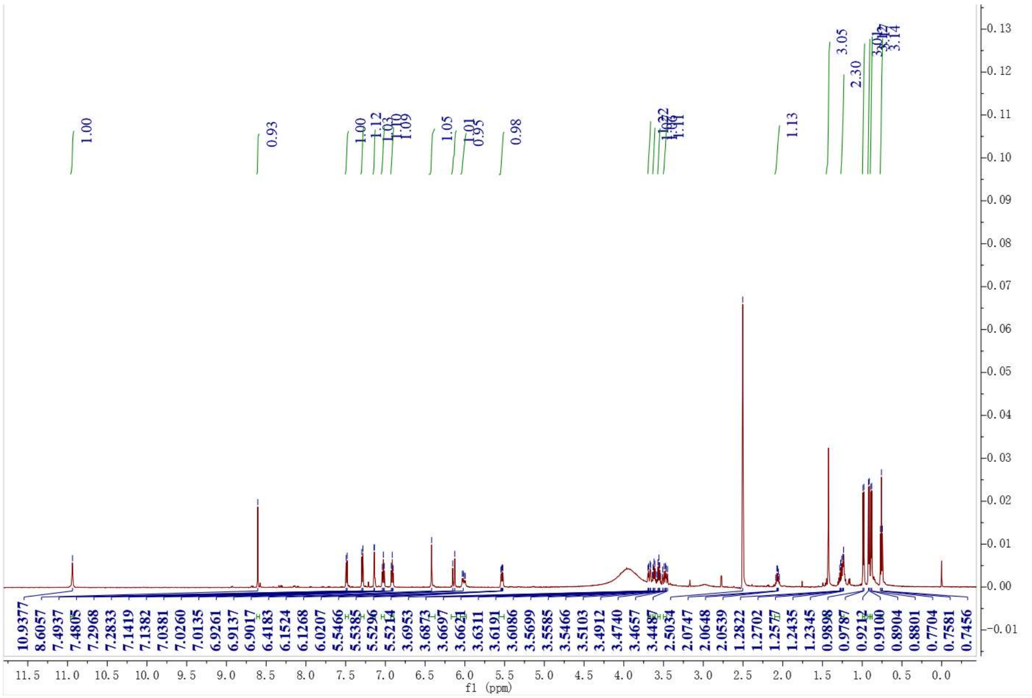

Figure S38. HRESIMS spectrum of compound **7**.

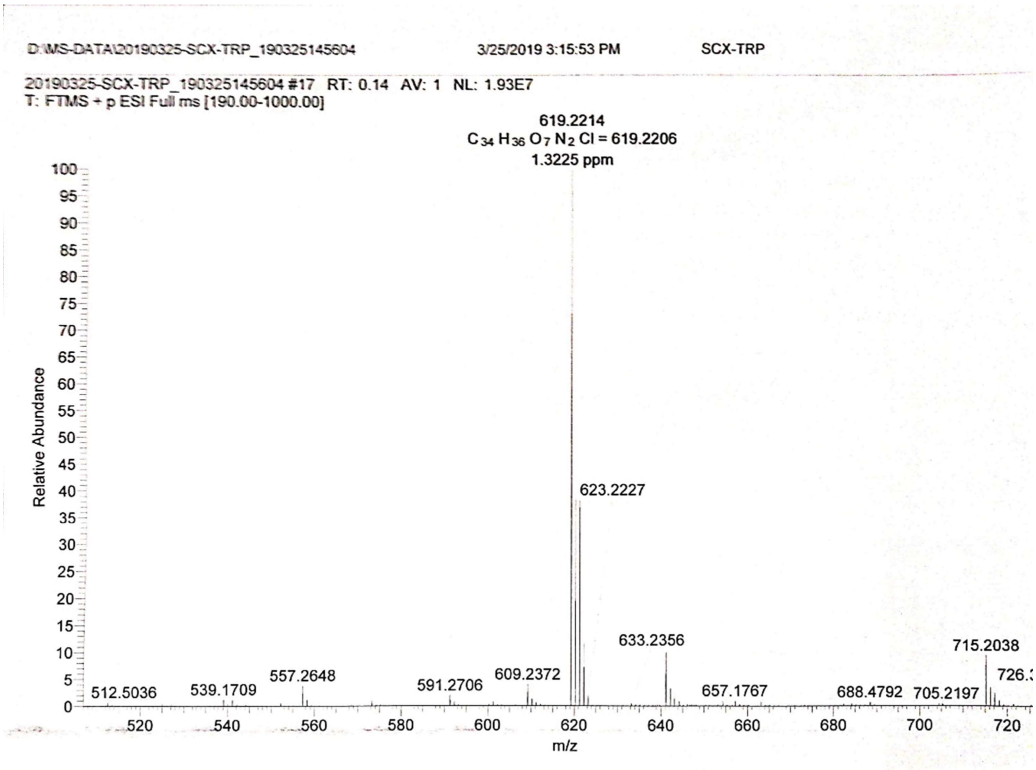

Figure S39. ^{1}H NMR (600 MHz, DMSO-d_6) spectrum of compound **8**.

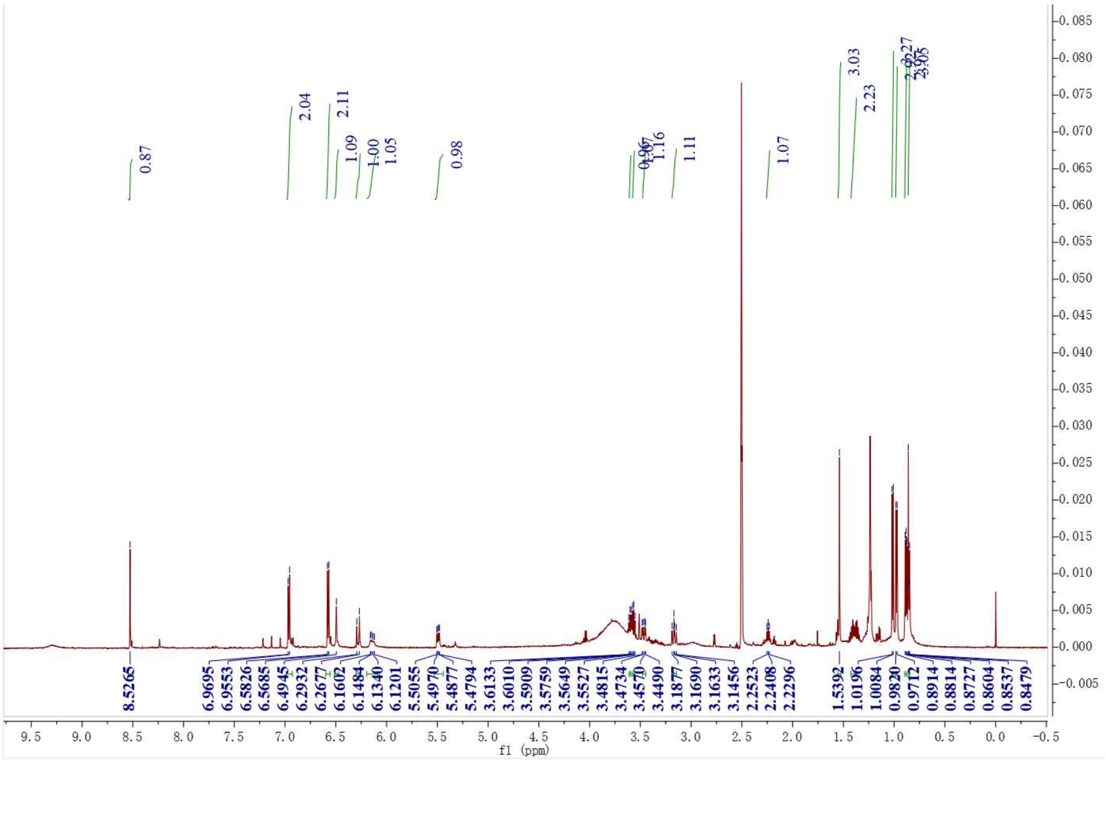

Figure S40. HRESIMS spectrum of compound **8**.

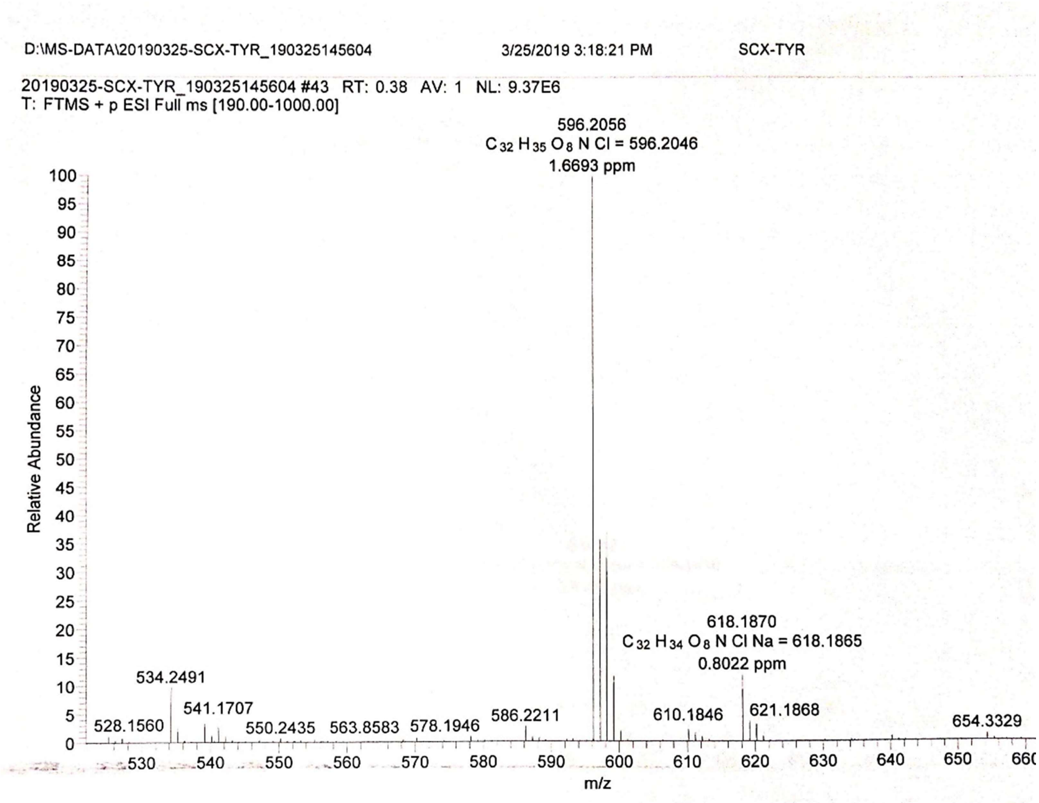

Figure S41. ^{1}H NMR (500 MHz, DMSO-d_6) spectrum of compound **9**.

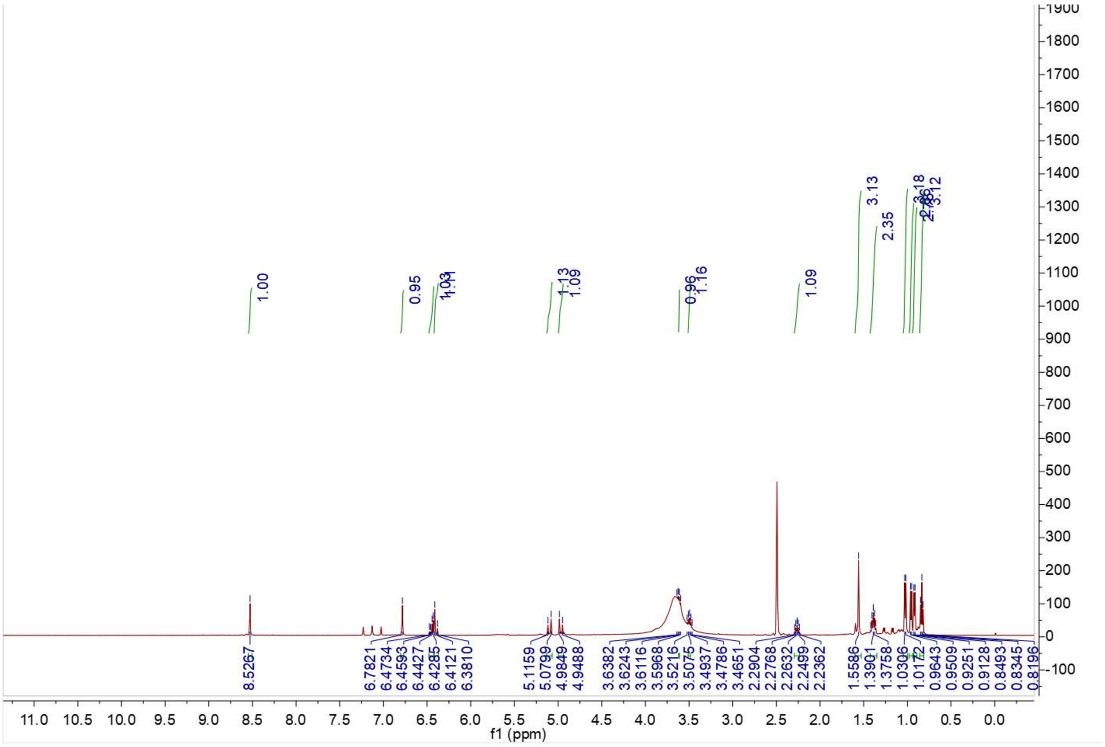

Figure S42. HRESIMS spectrum of compound **9**.

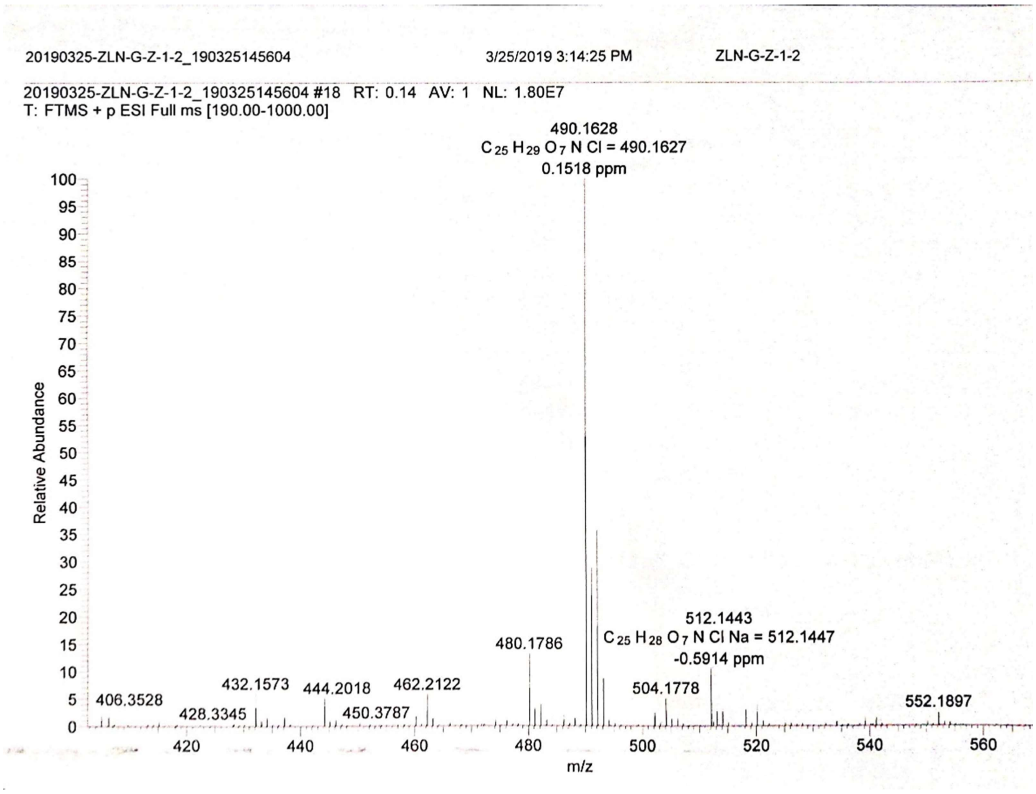

Figure S43. ^{1}H NMR (600 MHz, DMSO-d_6) spectrum of compound **10**.

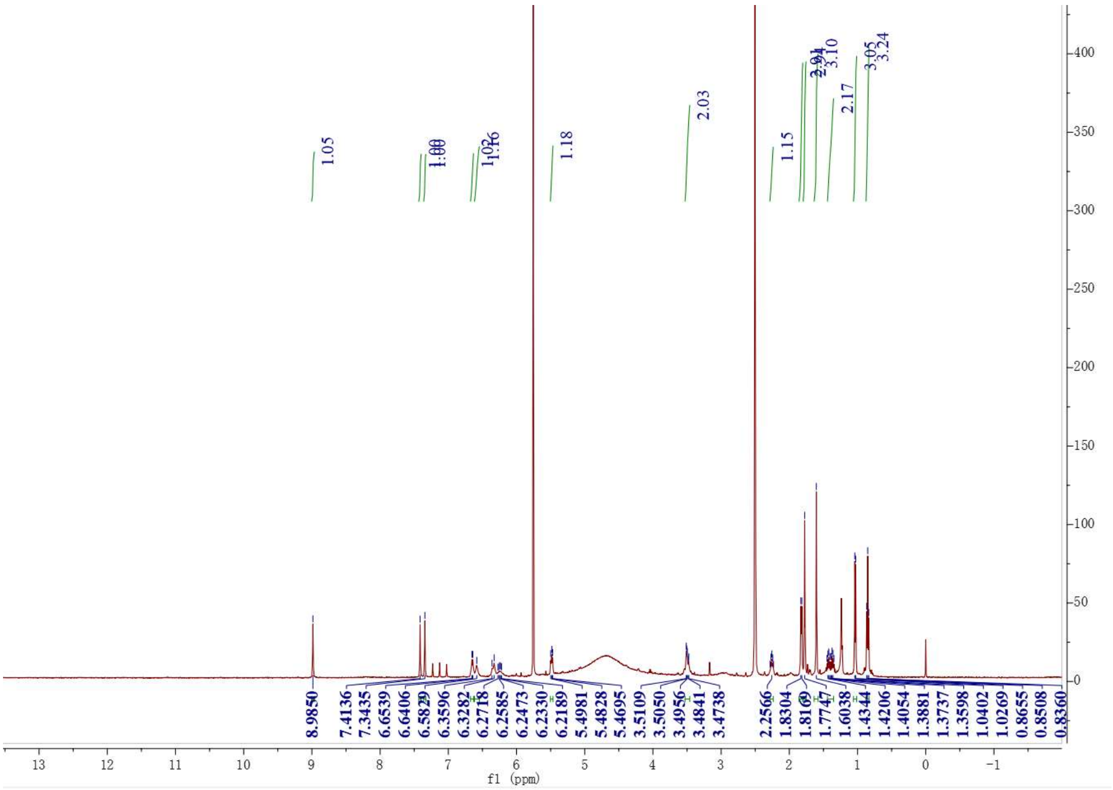

Figure S44. HRESIMS spectrum of compound **10**.

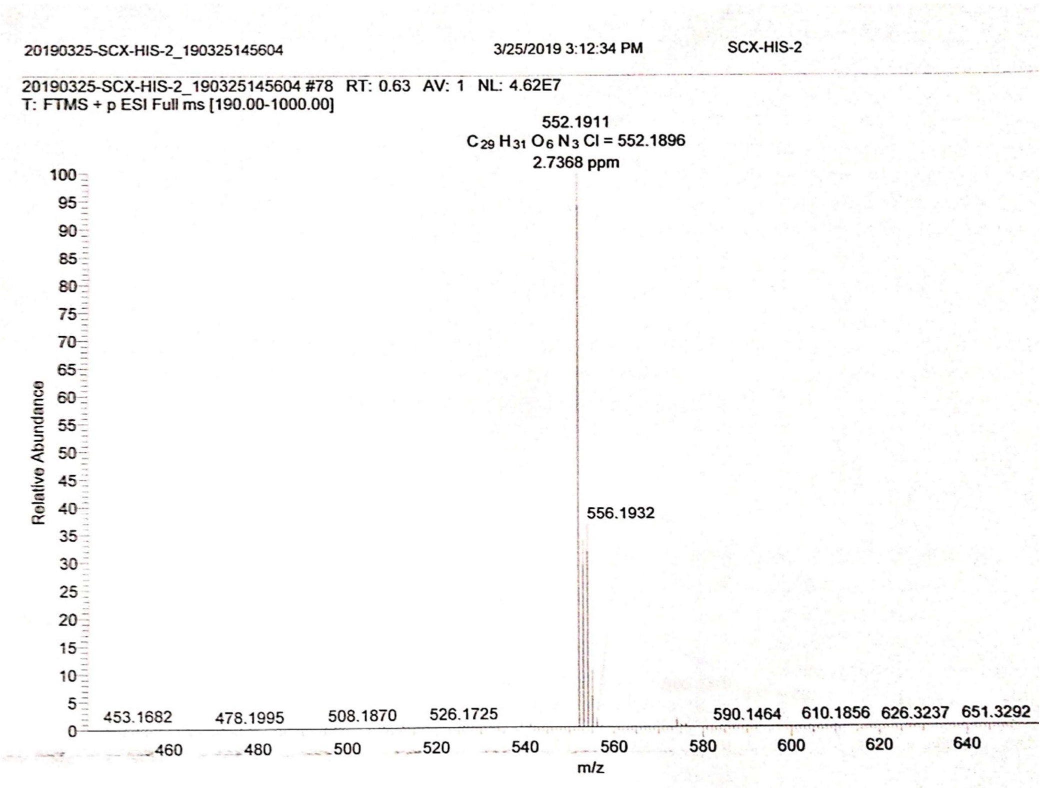

Supplement: Supplementary file 1 [file marinedrugs-17-00253-s001.pdf]
